# Pandemic influenza control in Europe and the constraints resulting from incoherent public health laws

**DOI:** 10.1186/1471-2458-10-532

**Published:** 2010-09-03

**Authors:** Robyn Martin, Alexandra Conseil, Abie Longstaff, Jimmy Kodo, Joachim Siegert, Anne-Marie Duguet, Paula Lobato de Faria, George Haringhuizen, Jaime Espin, Richard Coker

**Affiliations:** 1 Centre for Research in Primary and Community Care, University of Hertfordshire, College Lane, Hatfield, Hertfordshire, AL10 9AB, UK; 2 Communicable Diseases Policy Research Group, London School of Hygiene and Tropical Medicine, Keppel Street, London, WC1E 7HT, UK; 3 Technische Universität Dresden, Mommsenstraße 13 01069 Dresden, Germany; 4 INSERM 558 Unit/Centre Hospitalier Universitaire de Toulouse, 3 Centre Hospitalier Universitaire, Toulouse, France; 5 Escola Nacional de Saúde Pública, Universidade Nova de Lisboa, Avenida Padre Cruz 1600-560 Lisboa, Portugal; 6 National Institute for Public Health and the Environment, Centre for Disease Control, RIVM, Ant. van Leeuwenhoeklaan 9, PO Box 1, 3720 BA Bilthoven, The Netherlands; 7 Escuela Andaluza de Salud Pública (Andalusian School of Public Health) Campus Universitario de Cartuja, 4 Apdo de correos 2.070 Granada 18080, Spain

## Abstract

**Background:**

With the emergence of influenza H1N1v the world is facing its first 21^st ^century global pandemic. Severe Acute Respiratory Syndrome (SARS) and avian influenza H5N1 prompted development of pandemic preparedness plans. National systems of public health law are essential for public health stewardship and for the implementation of public health policy[1]. International coherence will contribute to effective regional and global responses. However little research has been undertaken on how law works as a tool for disease control in Europe. With co-funding from the European Union, we investigated the extent to which laws across Europe support or constrain pandemic preparedness planning, and whether national differences are likely to constrain control efforts.

**Methods:**

We undertook a survey of national public health laws across 32 European states using a questionnaire designed around a disease scenario based on pandemic influenza. Questionnaire results were reviewed in workshops, analysing how differences between national laws might support or hinder regional responses to pandemic influenza. Respondents examined the impact of national laws on the movements of information, goods, services and people across borders in a time of pandemic, the capacity for surveillance, case detection, case management and community control, the deployment of strategies of prevention, containment, mitigation and recovery and the identification of commonalities and disconnects across states.

**Results:**

Results of this study show differences across Europe in the extent to which national pandemic policy and pandemic plans have been integrated with public health laws. We found significant differences in legislation and in the legitimacy of strategic plans. States differ in the range and the nature of intervention measures authorized by law, the extent to which borders could be closed to movement of persons and goods during a pandemic, and access to healthcare of non-resident persons. Some states propose use of emergency powers that might potentially override human rights protections while other states propose to limit interventions to those authorized by public health laws.

**Conclusion:**

These differences could create problems for European strategies if an evolving influenza pandemic results in more serious public health challenges or, indeed, if a novel disease other than influenza emerges with pandemic potential. There is insufficient understanding across Europe of the role and importance of law in pandemic planning. States need to build capacity in public health law to support disease prevention and control policies. Our research suggests that states would welcome further guidance from the EU on management of a pandemic, and guidance to assist in greater commonality of legal approaches across states.

## Background

Emerging infectious diseases pose global challenges to human health protection. SARS and the emergence of avian influenza H5N1 galvanized political and public health communities to strengthen international, national and local preparedness and response capacities, and the emergence of H1N1 influenza has tested those responses. The International Health Regulations 2005 (IHR) represent an important international commitment to strengthening global capacity and acknowledge that law is part of the public health armamentarium underpinning cooperative national and international responses[2]. Whilst global disease surveillance capacity, in particular, has been considerably strengthened through the IHR, management of disease outbreaks, including pandemics, remains grounded in notions of national sovereignty. The same can be noted with regard to European co-operation, where regulations concerning surveillance and early warning are drafted with full respect for domestic law[3-6]. Harmonization of public health laws is not considered to be within the competence of the EU[7]. National systems of public health law are essential for influenza pandemic control, and international coherence will contribute to effective regional and global responses[1].

We report here results from a three year study analysing whether public health laws across the European Union, Croatia, Turkey, Iceland, Liechtenstein and Norway are 'fit for purpose', whether they are coherent with states' strategic national preparedness plans, ways in which laws differ and whether these differences are likely to be important from a public health perspective.

## Methods

### Participant states

The research focus was all European Union countries and the neighbouring countries of Croatia, Turkey, Iceland, Liechtenstein and Norway.

### Scoping exercise

A scoping exercise identified thematic issues. We conducted a literature review including reports from pandemic influenza simulation exercises, national strategic and operational plans[8,9], regional preparedness documents and research publications on public health law in Europe. The review was limited to documents in French and English that were available in the public domain between 2003 and 2007. With support from a panel of public health and legal experts key intervention themes were identified that were linked to World Health Organization (WHO) phases (table 1)[10].

**Table 1:** List of selected public health interventions linked to WHO pre-pandemic and pandemic phases

Pre-pandemic stage (Phases 4 and 5)	Pandemic stage (Phase 6)
Screening and medical examination	Obligation to provide healthcare

Isolation and quarantine	Prioritisation of healthcare

Compensation	Personal protective equipment

Vaccination and prophylaxis	Distancing measures

Treatment and decontamination	Closures, isolation and evacuation of facilities

Restrictions of contacts	Restrictions of movements

Compulsory measures (within the country and in relation to persons in transit)	Vaccines

Criminal offences	Requisition of persons, premises and goods

Obligation to provide healthcare to persons in transit	Other staff issues

Obligation of conveyance operators and airport authorities	Burial of deceased persons

Border closure	Prisons

Repatriation	Communication

### Data collection

Data collection was two staged:

#### Survey

In consultation with public health and legal experts and WHO, a self-administered questionnaire was developed, piloted and finalised. The questionnaire addressed the temporal phases and public health interventions that were identified through the scoping exercise. It consisted of 114 open-ended and closed-ended questions that were framed around an emergent influenza pandemic scenario (see additional file 1). Respondents from each country with legal and public health expertise were identified through WHO, ministries of public health, screening of pertinent publications, and through the European Public Health Law Network website developed within the project to facilitate communication between experts with an interest in public health law http://www.ephln.org. Questionnaires were sent by email with follow up reminders to ensure a high response rate. On receipt of the completed questionnaires, and where state pandemic plans were available in English or French, we compared the questionnaire results of each state with measures proposed in the pandemic preparedness plan of that state.

#### 2. Review

##### Identification of country experts

Participants with expertise in law and public health were identified through the membership base of the project network, European Public Health Law Network http://www.ephln.org. In addition, participants were identified through contact with ministries of health, academic institutions, professional networks, announcements at conferences, and searching journals to identify authors with relevant expertise. The persons who completed the questionnaire were in all cases except one, the persons who attended the review workshops.

##### Workshops

Four workshops were held (London, Toulouse, Prague and Lisbon), with a participant from each state attending one workshop. Building upon information provided through the questionnaires, we explored how national state laws might assist or constrain public health interventions in the context of the influenza pandemic scenarios previously described through the survey instrument and also, with the emergence and global spread of H1N1v in early 2009, how contemporary national responses were impacted by current national laws. Respondents then analysed how differences between national legal systems and between specific laws might support or hinder regional responses to pandemic influenza, and the impact of national laws in the following areas:

• the movements of information, goods, services and people across borders in a time of pandemic;

• the capacity for surveillance, case detection, case management and community control;

• the deployment of strategies of prevention, containment, mitigation and recovery;

• the identification of commonalities and disconnects across states.

Workshops were conducted under 'Chatham House rules' to encourage openness and the sharing of information between speakers whilst preserving anonymity in the reporting of the results. Workshops were audio-recorded and transcripts were subsequently sent back to participants for verification.

### Analysis

As noted, we identified *a priori *emergent themes through the scoping exercise. Data from questionnaires and workshop reviews were organised according to the analytical categories. We adopted a 'framework' approach to analysis[11], consisting of five interconnected stages: familiarisation; identification of a thematic framework; indexing; charting and mapping; and interpretation.

### Ethics approval

Not required.

## Results

### 1. Questionnaire results

The questionnaire was completed by participants from 23 states (Austria, Belgium, Bulgaria, Croatia, Cyprus, Estonia, Finland, France, Germany, Hungary, Iceland, Ireland, Latvia, Lithuania, Malta, the Netherlands, Norway, Poland, Portugal, Slovakia, Slovenia, Sweden and Turkey). All states have laws addressing the prevention and control of communicable diseases. In nine states there are also emergency powers provided in legislation that include a pandemic in the definition of an emergency, whilst fourteen states will limit interventions in a pandemic to those provided for by public health laws. Six of the Schengen states' plans consider a pandemic to constitute a serious threat to public policy or internal security to justify reintroduction of internal border controls. The Schengen Treaties (1985) on free traffic of persons, since 1997 incorporated into EU Law, are applicable in 26 countries, including the non-EU countries Norway, Iceland and Switzerland, but excluding the UK, Ireland, Bulgaria, Cyprus and Romania.

Ten states have laws that would authorise border closure in a pandemic.

Table 2 provides some examples of the range of measures provided across those states for which we have completed questionnaire results:

**Table 2:** Examples of pandemic measures with legal underpinning.

Measures	Out of 23 states
Reporting duties in relation to communicable disease	23

Reporting duties specific to human influenza	15

Compulsory screening	13

Compulsory isolation	17*

Compulsory quarantine	12*

Compulsory vaccination	9

Compulsory treatment	17

Provision of healthcare to an EU national resident in their state	18

Provision of healthcare to a visitor from an EU member state	18

Provision of healthcare to a visitor from outside Europe	16

Requisition of persons	16

Authorise unlicensed staff to be requisitioned to perform medical acts in a pandemic	7

Obligation of a worker to work in a pandemic	7

Requisition of premises	16

Requisition of goods	14

Compensation authorised for requisition of premises	10

School closures	20

Prohibition of mass gatherings	20

*This figure includes those states that intend to use this measure for 'listed' diseases. Even where influenza is currently not in the 'listed' category, the process of listing is simple and the intention is for the power to apply to pandemic influenza, once listed.

The organizational structure of governments differs across European states as a consequence of cultural factors and differences in legal system. Seven state rapporteurs in the project responded that their state operated on the basis of a federal or quasi-federal system: Austria, Belgium, Germany, Portugal, Spain, Sweden and the United Kingdom. See Table 3.

**Table 3:** Response to questions on devolution of powers

Questions	Yes	No
Do laws regulating public health fall within the devolved powers of regional governments?	Germany, Austria, Belgium, Portugal	Bulgaria, Turkey, Croatia, Poland, Cyprus, Ireland, Sweden, Estonia, Netherlands, Hungary, Iceland, Latvia

Does your country have communicable disease legislation/laws at national level?	Bulgaria, Germany, Turkey, Hungary, Poland, Austria, Cyprus, Finland, Malta, Ireland, Sweden, Belgium, Estonia, France, Netherlands, Slovenia, Slovakia, Iceland, Lithuania, Portugal, Norway, Latvia	

Does your country have communicable disease legislation/laws at regional level?	Germany, Austria, Belgium, Iceland	Bulgaria, Turkey, Hungary, Poland, Cyprus, Malta, Ireland, Sweden, Estonia, France, Netherlands, Slovenia, Slovakia, Hungary, Portugal, Lithuania, Norway

Does your country have communicable disease legislation/laws at local level?	Germany, Austria, France, Iceland	Bulgaria, Turkey, Hungary, Poland, Cyprus, Malta, Ireland, Sweden, Belgium, Estonia, Netherlands, Slovenia, Slovakia, Hungary, Portugal, Lithuania, Norway

In follow up research, the national pandemic plans of the five federal or quasi federal states where plans were available in English were analyzed, examining the distribution of pandemic planning roles and responsibilities between national and regional governments. See Table 4.

**Table 4:** Distribution of powers in federal or quasi federal states

	Structure	National Responsibility	Regional Responsibility	Summary
**Austria**	9 regions	Planning and co-ordination Situation monitoring Assessment Communication	Planning and co-ordination Situation monitoring Assessment Communication Overall responsibility for health care Responsibility increases in Phase 5	Regional involvement. Mainly decentralised

**Germany**	16 regions	Planning and co-ordination Situation monitoring Assessment Communication	Pandemic preparedness Strategic action by health system	Mainly centralised

**Spain**	17 autonomous regions or communities and 2 autonomous cities	Planning and co-ordination Situation monitoring Assessment Communication Reducing the spread of diseases	Planning and co-ordination Situation monitoring Assessment Communication Reducing the spread of diseases Responsibility increases as of Phase 3	Close involvement of Regional governments co-ordinated by National government

**Sweden**	21 provinces 290 municipalities	Planning and co-ordination Situation monitoring Assessment Communication Reducing the spread of diseases Guidance for health systems Management of health system Provision of a knowledge base	Planning and co-ordination Reducing the spread of diseases Contingency plans Provision of health care and medical services Distribution of vaccine and antiviral drugs	Mixed - response between National and Regional

United Kingdom*	4 regions	Four national health systems with a national Health Protection Agency Co-ordination and direction Strategy and activation of plan	Delivery Planning Support of National response	Mixed - response between National and Regional

* The rapporteur representing the United Kingdom did not return the completed questionnaire, so questionnaire results do not include information on devolution of powers in the UK. We did however include the UK in our own follow-up research.

### 2. Preliminary analysis of coherence between laws and plans

We were able to undertake a preliminary analysis to compare laws and plans in 11 states where plans were available in English or French. In only two of these states were all the measures proposed in plans supported by specific legal authorisation. In some states such as Belgium and France, however, there are general powers enabling any proportionate measure for a public health purpose. In other states, such as Ireland, there is a broad power to make regulations to prevent the spread of infectious disease and to treat people suffering from infectious disease that could authorise measures such as compulsory examination and treatment. Examples of proposed pandemic measures not supported by law included the use of unlicensed drugs, vaccines and health workers, closure of borders, control of media information and reporting, obligation of workers to work where there was risk to health and safety, compulsory isolation, quarantine, vaccination and prioritisation of people in access to vaccines and antivirals.

Plans of some states, for example Estonia, recognised the need to ensure a legal framework for measures while plans of most states made no mention of legal underpinning.

### 3. Review

Twenty-four countries were represented at review workshops (Austria, Belgium, Bulgaria, Croatia, Cyprus, Czech Republic, Estonia, Finland, France, Germany, Hungary, Iceland, Ireland, Latvia, Lithuania, Malta, the Netherlands, Norway, Poland, Portugal, Slovenia, Sweden, Turkey, and United Kingdom). Legal systems within Europe range from common law states (England and Wales, Ireland, Malta, Cyprus), to French style civil law states, German style civil law states and Scandinavian legal systems, to former Soviet Union states and one state, Turkey, with some influence from Islamic legal culture. These different legal systems may result in different understanding of what constitutes 'law'[12], and this was explored in the workshop discussion. Some states have laws, such as those in England and Wales and in Estonia, that are detailed and prescriptive. These laws tend to rely on lists of notifiable diseases, and public health measures can only be undertaken in relation to listed diseases. In other states public health laws are broadly framed allowing for the significant exercise of discretion. In Cyprus there are no specific laws governing measures such as quarantine but the Council of Ministers has powers to take all necessary measures. New laws in France allow the Minister of Health to take any measures that are proportionate to protect the public health.

Where laws give broad discretion to act, principles such as those of proportionality or precaution are likely to form part of the law. In Slovenia the principle of proportionality has been written into the Constitution and has since been developed by the Constitutional court. The 2004/2005 Constitution in France incorporates the precautionary principle. The new legislation of the Netherlands is built upon the principle of precaution requiring a risk assessment before measures are taken.

States are at different stages of development of communicable disease legislation. Some states are reliant on nineteenth century laws that have undergone some updating for IHR compliance. Other states have new laws designed with pandemic diseases in mind. Some states operate their public health laws at national level while in others with devolved systems regional laws are more important. In most states, laws relevant to pandemic influenza are contained in dedicated public health laws, but in some states other legislation and case law must also be included in the body of disease control law. In some states (for example, Ireland and Belgium) the Constitution limits measures that can be taken, providing greater opportunities for individuals to challenge public health interventions. In all states the European Convention on Human Rights (ECHR) is considered to limit the measures that can be taken under public health legislation, though not necessarily measures taken under emergency powers legislation.

Across states there are differing approaches to the status of pandemic preparedness plans. In Belgium and Slovenia, pandemic preparedness plans have the force of law in terms of both public perception and practice, whereas in other states measures proposed in the plan depend on legal underpinning. The relationship between laws and pandemic plans was not clear in many states. Comments by participants included, 'the (survey) questions were difficult to answer because of the lack of clarity between laws/decrees and the national preparedness plan'; 'the national preparedness plan was drafted without legal implications in mind at first'; and 'day to day practice is not aligned with the legal framework'; 'there is a gap between theory and practice. If there were a challenge to an exercise of a power, the court is likely to rule that it was up to the government to decide...'; 'There is a lack of clarity on competences and responsibilities, and the relevant bodies in charge.' In some states it was considered that legislation was more developed than the preparedness plan, and in others that the plan was well defined but that underpinning public health legislation was not sufficiently developed.

There are also differences in the extent to which emergency powers can be drawn upon in a pandemic. While emergency powers in France and Belgium apply only to war, in Slovenia, Hungary, Latvia and Lithuania emergency legislation specifically provides for communicable diseases and there are also emergency powers provisions in the contagious diseases legislation. In the Netherlands there is emergency powers legislation applicable to a pandemic, in exceptional circumstances and on a decision of the cabinet. In Estonia, two statutes provide emergency powers. Both apply to a pandemic and it is envisaged that they will be used. In the UK civil contingency powers play a role in pandemic strategy. The intention to use emergency powers is significant in that emergency powers may allow for greater intervention, with possible derogation from the European Convention on Human Rights[13].

### 4. Thematic coherence

Discussion in the workshops was facilitated in accordance with the predetermined themes.

#### a. Movement of people, goods and information

There is no common approach across the states represented as to the control of movement of people. Some states, such as Sweden and Ireland, envisage no restrictions. States such as Cyprus that are not signatories to the Schengen Agreement have the option to close borders against travellers from within Europe, and other states such as Slovenia and Estonia have emergency powers applicable to pandemic disease which might authorise border closures.

There are laws in some states to authorise restrictions on movement of goods, mostly on incoming rather than outgoing goods. Legislation in Malta and Cyprus allows for the stopping of movement of goods in and out of the country on public health grounds.

In relation to information, most states have incorporated the EU legislation on Data Protection[14]. Some states, Sweden for example, have passed laws to authorise the provision of public health data to public health authorities both within the state and to the EU and WHO.

#### b. Surveillance, case detection and management, community control

In relation to surveillance of influenza, there are variations in duties of notification across states. Most states have laws imposing on health professionals duties to disclose to specified public health authorities information on suspected or confirmed cases of disease. However in some states these laws may only come into play where the disease is listed as notifiable (for example in England and Wales, Ireland and Estonia).

There are differences in powers of compulsory screening and medical examination across states. While some states' laws authorise powers of compulsory treatment and compulsory vaccination, these measures are prohibited by laws in other states. In most states there is a capacity for community control such as prohibition of gatherings and school closures, specifically provided by communicable disease or other legislation such as education laws, or possibly at the discretion of a public health authority or by means of exercise of emergency powers.

There are differences in accessibility to healthcare resources across European states, with some states prepared to provide healthcare to non-citizens, such as Lithuania, where undocumented immigrants have access to free necessary healthcare. While some states, such as Malta, intend to continue to allocate healthcare on clinical grounds, where resources are limited states may use emergency powers to control movements of goods and people so as to prioritise state citizens in the allocation of health benefits.

#### c. The impact of laws on deployment of strategies of prevention, containment, mitigation and recovery

Pandemic plans set out strategies of prevention, containment, mitigation and recovery but workshop participants noted an insufficient link between pandemic plans and public health laws, potentially limiting the effectiveness of those strategies. Interventions proposed in some plans lack legal underpinning. Comments of workshop participants included, 'the preparedness plan mainly addresses health services. What is lacking is a link with the public authorities responsible for handling the pandemic'; 'public health authorities responsible for handling an outbreak have little knowledge about their role and how to prepare for it. There is confusion about who is competent'; 'there is a gap between the content of the preparedness plan and their awareness of their responsibilities'; 'there is a gap in planning in relation to coordination. For example people are not clear on their role in quarantine'; 'it is not clear how to implement some compulsory measures decreed by the Minister such as mask wearing in public. How do we oblige people to respect and comply with these measures?' and 'the infectious disease legislation gives some powers to the Minister to enforce the regulations but this is not actually done.' It was noted in relation to one state where laws gave powers to a public health authority: 'but this is premised on the assumption that they know what to do. It would be better if there were some directions/legal framework'.

Even where plans and laws are in place, clarity is lacking regarding which body is responsible for specific interventions and as to the organisation and management of pandemic planning. It was commented in the workshops that 'preparedness plans seem to assume that powers already exist', and 'it seems to have been assumed that the IHR 2005 would have direct effect, like a treaty. Some officials think the IHR 2005 are sufficient on their own to be considered as law, which is obviously not the case.' Several participants identified problems arising from a lack of expertise in public health law in their state, inhibiting implementation of pandemic planning. The lack of public health law expertise was even more significant in states where public health powers were not detailed in legislation. It was agreed by workshop participants that there is a need for education, training and research on the role of law in public health in Europe. One workshop participant commented, 'There is a definite need for public health law training for medical personnel and there are not enough courses available', and another, 'Health professionals do not have a sufficient understanding of how government departments such as the Ministry of Health are organised.' Other comments included, 'There is a need for more public health law expertise, as this issue is not well addressed within Europe'; 'There is little training in public health law. Law faculties and medical faculties keep very separate'; and 'Public health practitioners are not aware of public health laws.'

#### d. Commonalities and disconnects across states

Disease notification duties were generally common across states. Although not all states specifically required notification of influenza, there were powers to make influenza notifiable. Commonality in notification duties is not surprising, despite the differing systems of disease notification across states, as the IHR along with WHO and EU surveillance systems require harmonised reporting of pandemic disease notification. There was some commonality across states in powers of social distancing, although again these were not always specified in laws and in some states would require authorisation by a political or public health authority. There was less commonality in relation to powers of compulsory screening, examination, vaccination and treatment. While some states' laws authorise compulsory vaccination and treatment, other states' laws prevent vaccination or treatment without consent. In Lithuania a person can only be detained for the purposes of treatment, with a focus on individual benefit rather than public health, while in other states, such as England and Wales, a person can be detained but cannot be compulsorily treated. There were also differences in access to healthcare and in the obligation of healthcare workers to work in a pandemic. Project participants were concerned by the consequences of these differences for the movement of persons across Europe.

A majority of participants suggested a need for greater guidance in the management of a disease pandemic. It was commented that 'a European response would be much more practical and easier, rather than states making their own decisions'; 'it would be good to have uniform guidelines to avoid medical tourism, for example', and 'if you have designated points of entry under the WHO, it would be helpful to have EU advice and commonality on the understanding of what is a designated point of entry. If people are coming from outside the EU and then moving around within the EU, it would help to have some commonality of rules'. One participant noted, 'there are economic considerations. Individual states are reluctant to be the first to take measures such as contact tracing, for economic reasons. It would be better if states decided together'. Others noted, '-It would be better to have laws harmonised across Europe. There ought to be better co-ordination of plans, however, countries should not be forced to follow others' plans'; and 'Politically the EU is trying not to be too interventionist. But we do need some coordination as there are no border controls. We also need coordination on issues of distribution of resources across Europe'; 'The EU should keep states posted on changes in the legal environment'; and 'There is a need for coordination and guidance from the EU in terms of management, but this may be difficult, thinking of refugees'. But further EU involvement would not be useful for all states: 'We have no expertise in public health law. But the public health specialists are knowledgeable on the relevant law. As to EU involvement, WHO and ECDC are more helpful... than the EU'.

## Discussion

In a democratic system that recognises international and European human rights conventions, interventions that infringe liberties must be enshrined in law. While public health policy and pandemic planning might propose measures beneficial to the public health, those measures cannot be applied without legal underpinning. The results of this study show differences across European states in the extent to which national pandemic policy and pandemic plans have been integrated with public health laws. There are differences in the legal status of pandemic plans; in some states plans have for all practical purposes the status of law, and in others plans have no legal authority.

A consequence of the disconnect between plans and laws is lack of clarity as to the responsibility and competence of public health authorities. Only in two states were lines of command thought to be clear, coordinated and detailed. One role of law is to provide an inviolable framework for policy and the application of powers. Where pandemic plans have been prepared independently, and in ignorance or neglectful, of law, and where the legal framework has not been updated to reflect plans, then confusion is inevitable. A common theme in workshop discussions was the lack of clarity within states and across states as to the authority responsible for the management of different aspects of disease control. Authorities in one state are not always clear which body is their equivalent in other states, and whom to contact. This is a particular problem in states where powers are devolved to regional or local levels. For example in Sweden, where the system is decentralised and law places responsibility at regional/local level, there are 21 regional preparedness plans with their own responsible public health bodies. Further work needs to be undertaken on making clear pandemic responsibilities and competences. This is an issue of concern, given the lead-in time we have been given for a pandemic resulting from SARS and avian influenza H5N1. It is hoped that the H1N1v pandemic, a pandemic associated with limited morbidity and mortality, will provide an opportunity to establish responsibilities and hierarchies in management.

While some states have passed new public health legislation addressing contemporary understanding of public health risks, many states have public health laws that originate in the nineteenth century. In some cases attempts have been made to amend laws in recognition of IHR obligations and pandemic planning, without addressing the outdated science and jurisprudence that underlay old legislation, resulting in an inaccessible collection of uncoordinated and unconsolidated laws. Lack of public health legal expertise across Europe compounds this inaccessibility. While rapporteurs reported an intention by their states to make national laws compliant with the IHR by 2012, there appears to be insufficient understanding in many states of the role of the IHR, of what is required in the way of compliance in Annex 1 of the IHR and of the relationship of the IHR to state plans and laws, despite guidance on legislative compliance provided by the WHO[15]. Our results suggest that lack of understanding of laws by persons working in public health has contributed to lack of coherence between the IHR, plans and laws in some states. As has been noted elsewhere[16] the IHR (2005) like its predecessor regulations, relies on non-binding recommendations and guidance ('soft law') rather than on legal obligations, such that compliance by signatory states cannot be assured.

In some states laws that might be needed in a pandemic are not yet drafted, and the intention is to draft these laws when they are needed. It is essential that laws authorising public health powers be passed and disseminated in advance of a pandemic and that reactive laws are not drafted in times of crisis. In states where public health law is broadly framed leaving much to the discretion of public health authorities, and in states where plans rely on the exercise of emergency powers, hurriedly drafted laws might be difficult to challenge on constitutional and human rights grounds.

Workshop discussion suggested that there is an argument for greater involvement of the European Union in the management of pandemic disease, in the form of recommendations and guidelines. Participants argued for more input on disease management from the EU. There was concern that with disparate laws, states will respond differently in their preparedness to carry out measures such as contact tracing or the passing on of information on travelers. There is also discrepancy in healthcare resources across states within Europe, and a lack of clarity on strategies to cope with consequent movements of populations seeking care. Different approaches to access to healthcare across Europe might result in movement of populations.

### Limitations of study

The project, scenario and questionnaire were designed with H5N1 influenza in mind, and the first two workshops considered plans and laws on this basis. However by the time of the two final workshops, the H1N1 pandemic was well established, altering the focus of some aspects of the discussion. For example in the second two workshops, participants were concerned about the feasibility of legal reporting obligations where disease was so widespread, and possible conflict between national laws on disease reporting and WHO advice. The emergency of pandemic H1N1 halfway through the study provided useful data on the practical application of plans and laws and on amendments to plans and laws necessitated by the experience of a pandemic. Discussants in the final two workshops had the advantage of assessing the application of plans and laws in that context.

While some of the experts representing states were government appointed or recommended, some were self-selected. The appropriateness of these experts was verified by means of their publications and their professional history. The challenge of finding experts is in itself an important finding of the study, highlighting the need for capacity development and coordination in this field.

Some workshop participants were trained in law, some in public health and some in both. The differing language of the disciplines of law and public health resulted in different interpretations of the questionnaire questions and different understandings of some legal terminology.

We were unable to recruit participants from seven states, primarily because we were unable to identify persons with appropriate public health law expertise in those states.

We were reliant on the expertise and knowledge of the state rapporteurs for information on their states. Where possible we cross checked responses but in many instances this was not possible as laws and pandemic plans were in languages in which we have no expertise.

The scope of the project focused on the extent to which national laws supported or constrained the implementation of pandemic preparedness plans. It was not an objective of the project to evaluate national public health legislation against the International Health Regulations (2005), although issues in relation to IHR compliance emerged in workshop discussion. Such an evaluation would be a worthwhile area of further research.

## Conclusions

There are significant differences in legislation and in the legitimacy of conduct across states in Europe. In some states pandemic plans are part of the law and in others not. In some states disease measures are clearly specified in advance, and in others measures are to be determined where the need arises. States differ in the range and the nature of intervention measures authorized by law, the extent to which borders will be closed to movement of persons and goods during a pandemic, and access to healthcare of non-resident persons. Some states propose use of emergency powers that might potentially override human rights protections while other states propose to limit interventions to those authorized by public health laws. These differences could create a problem for European strategies.

Differences across Europe in legal systems, in the breadth in which public health law is framed, in the level of discretion given to ministries and public health authorities and the extent to which emergency powers are to be used in a pandemic, mean that comparison and evaluation of efficacy of laws across European states is a difficult task. The results of our research suggest that states would welcome further guidance from the EU on management of a pandemic, and guidance to assist in greater commonality of legal approaches across states. There will be ramifications of incoherence of laws across states for movement of populations, transportation of drugs, access to healthcare and for human rights and data protection. There is a need for further analyses to determine the public health implications of differences in laws, and whether regions beyond Europe are more coherent in their legal responses to pandemic influenza.

There is a dearth of expertise and training in public health law across Europe. Most pandemic planning in Europe is undertaken by public health practitioners with no input from persons with expertise in law, and workshop results suggest that there is limited understanding of the relationship between law and public health practice in the management of disease prevention and control. This suggests an urgent need for improved training in public health law in both the law and healthcare sectors.

## Competing interests

The authors declare that they have no competing interests.

## Authors' contributions

RM, RC, AL and AC contributed to the writing and review of this paper. All authors participated in the design of the study, and contributed to the original data and to the editing of the report. All authors have read and approved the final version of the manuscript.

## Funding

PHLawFlu was co-funded by the European Union. The funder had no role in the design, analysis, or interpretation of the study. The views expressed are those of the authors and do not necessarily reflect the position of the funding body.

## Pre-publication history

The pre-publication history for this paper can be accessed here:

http://www.biomedcentral.com/1471-2458/10/532/prepub

## Supplementary Material

Additional file 1 **Questionnaire consisting of open-ended and closed-ended questions addressing national laws in relation to public health interventions in an influenza pandemic**. Click here for file
