# Supplementary material for: Pandemic influenza control in Europe and the constraints resulting from incoherent public health laws
Source: BMC Public Health. 2010 Sep 3;10:532. doi: 10.1186/1471-2458-10-532 (PMC2945945; doi:10.1186/1471-2458-10-532)
Supplement: Additional file 1 — Questionnaire consisting of open-ended and closed-ended questions addressing national laws in relation to public health interventions in an influenza pandemic. [file 1471-2458-10-532-S1.DOCX]

**PHLAW/FLU PROJECT - SCENARIO WORKSHOP**

**Content**

[I- Briefing paper on Influenza for the workshop 2](#_Toc213731846)

[ii- Background to the workshop 4](#_Toc213731847)

[iii - Some general questions about your country’s public health law framework 5](#_Toc213731848)

[iv- narrative Part a - (pre-pandemic period: who Phases 4 and 5) 9](#_Toc213731849)

[IV- Legal questions - part A: Pre-pandemic period 12](#_Toc213731850)

[V- narrative Part B - (pandemic period: who Phase 6) 33](#_Toc213731851)

[VI- Legal questions - part B: Pandemic period 35](#_Toc213731852)

[VIII- Glossary 49](#_Toc213731853)

|  |  |
| --- | --- |
|  |  |
|  |  |
|  |  |

# I- Briefing paper on Influenza for the workshop

What are the differences between **avian influenza** and **human influenza**, and **seasonal influenza**  and **pandemic influenza**? ^[[1]](#footnote-1),^^[[2]](#footnote-2),^^[[3]](#footnote-3),^^[[4]](#footnote-4),^^[[5]](#footnote-5),^^[[6]](#footnote-6)^

**Avian influenza** refers to an infection caused by avian influenza viruses which primarily affects birds. Generally, the viruses circulate only between birds. This dynamic is referred to as *bird-to-bird transmission*.

However, on extremely rare occasions, avian influenza viruses undergo genetic changes and can infect other species, including humans. When this phenomenon occurs, *bird-to-human transmission* is observed. Under these circumstances, humans are infected through bird contacts only and cannot or rarely infect other humans. As long as birds remain the main agents of infection, influenza, the infection is characterised as “avian influenza”.

At a subsequent stage, it is possible that avian influenza viruses undergo further genetic changes. When this phenomenon occurs, avian influenza viruses develop the ability to transmit between humans. This is known as *human-to human transmission.* As long as small and large clusters of human-to-human transmission develop in populations, the infection remains known as avian influenza. However, once human-to-human transmission is sustained in populations, the infection will no longer be referred as avian influenza. It will be called pandemic (human) influenza.

**Human influenza** refers to an infection caused by human influenza viruses which affect and circulate in a sustainable manner in human populations. This dynamic is referred to as *human-to-human transmission*.

Human influenza infections can be categorised into two groups:

1. **Seasonal influenza** refers to an infection caused by human influenza viruses which circulate and affect populations on a seasonal basis. In Europe, this phenomenon occurs each winter. Influenza viruses at the origin of these seasonal epidemics are not completely new to humans and part of the population is immune to them. The infection is characterized by a sudden onset of high fever, myalgia, headache, cough, sore throat, and rhinitis. Most people recover within one to two weeks without requiring any medical treatment. However, in very young people, the elderly and individuals suffering from specific medical conditions, the infection may lead to severe complications. Under these circumstances, medical attention is required and death sometimes occurs.
2. **Pandemic influenza** refers to an infection caused by human influenza viruses which globally circulate and affect populations at irregular and sparse time intervals. In the twenty-first century, this phenomenon occurred at three points in time: 1918, 1957 and 1968.

Human influenza viruses that cause global epidemics- better known as pandemics - are completely new to humans, hence most people have little or no immunity. They often originate from avian influenza viruses. When this phenomenon occurs, avian influenza viruses may develop the ability to transmit between humans. This is known *human-to human transmission.* Once, *human-to human transmission* is sustained in populations, the viral source of the infection will no longer be referred an avian virus – it will be a human influenza virus.

|  |  |
| --- | --- |

# ii- Background to the workshop

This workshop is being held as part of the PHLawFlu project, which has received funding from the European Union in the framework of the Public Health Programme. The PHLawFlu project aims to explore how national laws might support or constrain pandemic human influenza preparedness across Europe. EU Generic Preparedness Planning assumes a degree of coherence of public health legal tools across European states. It is essential to determine the extent to which there is a commonality of public health laws across states, and to identify any gaps, inconsistencies, conflicts and critical problems between state legislation or laws in relation to responses to public health emergencies, and in particular in relation to state responses to a human influenza pandemic. To learn more about the project, see [www.ephln.org](http://www.ephln.org).

**Objectives of the scenario and the workshop**

The scenario and workshop focus on a fictitious pandemic influenza situation.

The purpose of the scenario is to give context to questions about public health laws which authorize exercise of disease control duties and powers in an influenza pandemic.

The objective of the workshop is to examine the public health laws (and any other piece of legislation, where applicable) relating to disease control in your state, and in seven other European states, in order to identify together any situations which might give rise to problems in the case of an influenza pandemic. Your workshop is one of four identical workshops, each workshop looking at the public health laws and suchlike of eight European states. The final output of the four workshops will be a profile of public health laws underpinning pandemic preparedness across Europe.

**Why participate in this workshop?**

It is essential to your country’s preparedness planning to be aware of functional coherence and incoherence of preparedness planning across the states which surround you. If states within Europe have different legal approaches to issues such as detention, border closure, compulsory vaccination etc., this may have implications for disease spread, for the movement of populations, or for the response of your country’s people to your disease control measures.

Your participation in this workshop will alert you to other states’ approaches to pandemic disease control, to assist your state in the management of disease control where there are differences between states. The workshop provides an opportunity to share good practice in the framing of public health laws, in order to improve the legal underpinning of good public health practice across Europe.

# iii - Some general questions about your country’s public health law framework

**Instructions for completing this questionnaire**

You have been chosen to participate in this research because of your expertise in your country’s public health laws. The aim of this introductory questionnaire is to give an overview of your country’s legal system and main public health legislation, **before** considering the more detailed disease scenario (sections IV and VI). Please complete this section and send it 3 weeks before the workshop. Please note that:

- We are aware of international and EU legislation forming part of national laws of the Member States^[[7]](#footnote-7)^ which addresses the issues covered by this scenario.
- There are no right or wrong answers to these questions. Some questions may not be relevant to your country. Please ignore these questions and proceed to the next question.
- In the **References** column, please give where possible the complete legal reference for each law; i.e. the **title** of each law, the **date of entry into force**, **where it is officially published, and the section, chapter** and **article** where applicable.
- Where your laws are in the **process of amendment**, please **complete the questionnaire on the basis of law currently in force**, but indicate in the **Comments** column where amendments are taking place and, where possible, the content of any proposed amendment.
- It may be that your country has laws addressing particular issues at national and regional and /or local level, in which case please indicate this in the **Comments** column.
- For countries with **Federal** legal systems, your answers may need to represent one jurisdiction within your country. Please indicate where possible, in the **Comments** section, in which jurisdiction the law is to be found. If possible, please also indicate if the law would be different in another jurisdiction within your country.

Definitions of terminology in ***italics*** can be found in the accompanying **Glossary,** which is also accessible online at: <http://ephlnglossary.blogspot.com/>

|  | **Questions** | | No | | Don’t know | yes | **References** | **Comments** |
| --- | --- | --- | --- | --- | --- | --- | --- | --- |
| 1 | Do you have in your country legislation or codified laws addressing communicable disease? | |  | |  |  |  |  |
| 2 | Do you have laws addressing communicable disease which are not found in legislation or codified form (where for example the source of law is common law, customary law, constitutional powers etc)? | |  | |  |  |  |  |
|  | a | If yes, please give examples: |  | | | | |  |
| 3 | Does your country have a federal legal system, whereby primary law-making powers are devolved to regional governments? | |  |  | |  |  |  |
|  | a | Do laws regulating public health fall within the devolved powers of regional governments? |  |  | |  |  |  |
| 4 | Does your country have communicable disease legislation/laws at: | |  | |  |  |  |  |
|  | a | national level? |  | |  |  |  |  |
|  | b | regional level? |  | |  |  |  |  |
|  | c | local level? |  | |  |  |  |  |
| 5 | As a member state of WHO, has your country made any reservation to the IHR 2005? | |  | |  |  |  |  |
| 6 | Have the IHR 2005 been incorporated into your national/federal legislation? | |  | |  |  |  |  |

|  | **Questions** | | No | Don’t know | Yes | **References** | **Comments** |
| --- | --- | --- | --- | --- | --- | --- | --- |
| 7 | Is your country a member of any Convention /Treaty/Agreement on *border control* (e.g. *EFTA, Nordic Agreement, Schengen* etc.)? | |  |  |  |  |  |
|  | a | If yes, please give details |  | | | |  |
|  | **Questions** | | No | Don’t know | Yes | **References** | **Comments** |
| 8 | If your country is a Member State of the Schengen agreement, does your preparedness plan make clear whether a pandemic will be considered a *serious threat to public policy or internal security* justifying reintroduction of its internal border control? | |  |  |  |  |  |
| 9 | Does your country have laws specifically addressing human health in relation to *avian* *influenza* (see Glossary definition)? | |  |  |  |  |  |
| 10 | Do your laws pertaining to *avian influenza* authorise any of the following compulsory preventive measures in relation to individuals working in an avian flu area (cullers, vets, farmers and families, etc.)? | |  |  |  |  |  |
|  | a | *prophylaxis* |  |  |  |  |  |
|  | b | *vaccination* |  |  |  |  |  |
|  | c | *restriction* on movement |  |  |  |  |  |
|  | d | restriction on work |  |  |  |  |  |
|  | e | other measures |  |  |  |  |  |

|  | **Questions** | | | No | Don’t know | Yes | **References** | **Comments** |
| --- | --- | --- | --- | --- | --- | --- | --- | --- |
| 11 | Does your country have laws which specifically address: | | |  |  |  |  |  |
|  | a | | *seasonal influenza*? (see Glossary definition) |  |  |  |  |  |
|  | b | | *pandemic influenza*? (see Glossary definition) |  |  |  |  |  |
| 12 | Does a disease pandemic fall within the definition of an emergency for the purposes of your country’s emergency powers legislation? | | |  |  |  |  |  |
| 13 | In case of a pandemic, will enacting of special emergency measures require authorisation by parliament? | | |  |  |  |  |  |
|  | a | If yes, is the authorisation required prior to such measures being taken? | |  |  |  |  |  |
|  | b | Can such measures be taken and then submitted for approval after a period of time? (Please specify the delay). | |  |  |  |  |  |
|  | c | Are such measures time limited such that they will need new authorisation after a set period of time? | |  |  |  |  |  |
| 14 | Does any agency/body have *delegated authority* to make emergency regulations or to provide emergency powers? | | |  |  |  |  |  |
| 15 | Is there an accessible and independent review and/or redress mechanism for the application of emergency measures? | | |  |  |  |  |  |

# iv- narrative Part a - (pre-pandemic period: who Phases 4 and 5)

**The emergence of HXNY in Europe and the development of first clusters of human cases**

**Background to today’s events**

During the months of ***November and December 2008***, the national authorities of ASIA1 have been investigating reports of unusually elevated rates of human sickness and death caused by an influenza-like illness in two distant villages located in the southern part of a province (both villages have a high density of poultry). An investigating team visited the two villages and collected samples of specimens both from poultry showing signs of sickness and from villagers who reported symptoms of influenza, and sent them to the national laboratory. The investigation revealed that, in the days preceding illness, all cases had been exposed to poultry. Subsequently, the national laboratory confirmed that most samples tested positive for avian HXNY. Hence, the team concluded that the villagers had acquired the infection through exposure with infected poultry and that it was very unlikely that they had acquired the virus through human-to-human transmission. It was announced that all poultry in the two villages under consideration were to be culled and that surveillance and health education measures would be intensified in the area.

At the ***end of January 2009***, the national authorities of ASIA1, through its national IHR focal point, confirmed to the World Health Organization (WHO) and to the Food and Agriculture Organization of the United Nations (FAO) that mass culling had successfully been carried out in the two villages and that no new animal or human case of avian influenza (AI) had emerged in the past two weeks. However, ***three weeks ago***, seven human cases of HXNY were detected in a village with low poultry density located in the northern part of the province. Since then, small clusters of human cases have emerged throughout the province and there has been sufficient supporting evidence to confirm that these cases were the result of human-to-human transmission. Consequently, the WHO has announced that the global alert has been raised to **Phase 4** and called on all countries to be extremely vigilant and to implement their action plans according to recent developments.

**EU2, Today, 30 March 2009**

It was revealed ***this morning*** that a man who had been admitted to a EU2 hospital yesterday night is suspected of being infected with HXNY. The man, who may become the first HXNY human case in Europe, is currently being kept in hospital for observation under strict isolation in the infectious disease unit. His history reveals that he has very recently returned from a visit in ASIA1, where he had been in contact with a sick relative who was potentially infected with human HXNY In EU2, the man lives with his wife. A nurse questions the man as to his activities during and after his business trip and asks him about the current health status of his wife. His condition is reported to the public health authorities. Specimens are collected from the man and sent to the national influenza centre (and subsequently to the WHO collaborating centre located in the United Kingdom). The man’s condition rapidly deteriorates and he subsequently dies.

The hospital’s chief physician, who worries that the man’s wife was exposed to HXNY, asks for biological samples to be taken from her immediately and for her to be monitored over the next 72 hours under strict quarantine in the hospital premises. The man’s wife refuses to comply with the proposed measures. The chief physician allows her to monitor her health from home and strongly advises her to meet with the unit’s health education nurse for a briefing on precautions to be taken by suspected HXNY carriers.

The laboratory confirms that the deceased man tested positive for HXNY. Soon after, EU2 commences to trace his recent contacts, including the crew and all passengers on his return flight from ASIA1. As a result, in EU2, there are now 220 individuals and their household contacts under quarantine or self-isolation at home, all taking a prophylactic course of antiviral medications (AV). The airline reports that a further 30 passengers connected directly to other flights: seven individuals flew on to your country and the others to EU1 and EU3. Consequently, EU2 communicates to your country and to EU1 and EU3 a list of individuals who may be at risk through contact with the deceased, and requests collaboration in tracing the passengers and in implementing appropriate early containment measures.

**Your country, 5 April 2009**

The public health authorities of your country actively commence searching for the seven passengers who flew to and entered your country, all of whom are nationals of your country, as they wish to isolate/quarantine them in hospital settings and administer to them a course of AV. One of the passengers, who lives alone and does not report any symptoms of influenza, agrees to take AV prophylaxis but refuses to have samples taken or be examined, and he declines quarantine measures. Another passenger, who was seated next to the man in the plane and who is feverish, is strongly suspected of being a HXNY case. He refuses to comply with all proposed measures as he says he must attend an important business meeting in a couple of hours. While attempting to trace the last passenger, a female teenager, the authorities are informed by a member of her family that she has been admitted to hospital suffering from severe respiratory problems. Samples taken from the teenager confirm she is infected with HXNY. She is the first HXNY case in your country.

**At the main airport of your country, 7 April 2009**

Your country implements entry screening at airports for passengers coming from ASIA1 and its neighbouring countries, who wish to enter your country. Passengers with symptoms or signs suggestive of influenza are initially isolated at the airport and then transferred to special treatment centres. Some passengers refuse to be screened and/or to comply with proposed measures which include isolation/quarantine, invasive and non-invasive medical examination, and antiviral therapy.

Two passengers, both who appear feverish and are reported to have been vomiting during the flight, arrive from ASIA2 and wish to connect to a flight to another European country. They are suspected by a member of crew to be infected with influenza. The airline on which they were flying notifies the airport authorities that there might be two cases of infection.

**In EU3 and in your country, 1 May 2009**

In S.E. Asia, larger clusters of human cases are forming and, today, WHO announce that the global pandemic alert level has been elevated to **Phase 5**. In the European region, the virus continues to spread (e.g. in your country, there are now 125 cases notified in ten different cities).

EU3, which still has no reported cases of the viral infection, announces that it will close **all** its borders to people wishing to enter or exit the country*.* It advises its citizens abroad to remain in their current location. This measure causes a storm of protests, particularly amongst EU3 nationals who wish to return to their homeland. Some nationals claim that they have a right to repatriation and a number of EU3 citizens wait at EU3 borders demanding to be readmitted. Your country decides to do the same. This decision sparks many concerns. For example:

- Mr L wants to leave your country and return to his home in EU3. He has no resources in your country.
- Mrs M who is on her way home to EU1, needs to drive through your country, but she is denied access to your country.

In EU3, all non-nationals unable to leave because the country’s borders are closed are refused access to healthcare services.

**END OF NARRATIVE PART A**

# IV- Legal questions - part A: Pre-pandemic period

**Instructions for completing this questionnaire**

Please note that:

- Some measures described in the scenario may have limited scientific support but they are nonetheless mentioned because some countries have described them in their protocols.
- There are no right or wrong answers to these questions. This study is simply a comparative study of proposed interventions and underpinning laws. Owing to the number of countries involved in this study, some questions may not be relevant to your country. Please ignore these questions and proceed to the next question.
- In the **References** column, please give where possible the complete legal reference for each law; i.e. the **title** of each law, **date of entry into force**, **where it is officially published, section, chapter** where applicable and **article**.
- Where **your laws** are in the **process of amendment**, please complete the questionnaire on the basis of law currently in force, but indicate in the **Comments** column where amendments are taking place and, where possible, the content of any proposed amendment.
- In the **Comments** column, please indicate where possible, by ticking the appropriate box, at which level the law was enacted: tick box **N** for law enacted at national level, box **R** for law enacted at regional level and box **L** for law enacted at local level. It may be that your country has laws addressing particular issues at several levels, in which case tick all the relevant boxes. If further explanation is needed, please comment in the **Comments** column.
- For countries with Federal legal systems, your answers may need to represent one jurisdiction within your country. Please indicate where possible, in the **Comments** section, in which jurisdiction the law is to be found. If possible, please also indicate if the law would be different in another jurisdiction within your country.
- Definitions of terminology in ***italics*** can be found in the accompanying **Glossary**.
- The events presented in this scenario are fictitious. We have not made assumptions as to the viral strain which might cause an influenza pandemic. Hence the viral strain is referred as HXNY.

|  | **Reporting** | | | | | | |
| --- | --- | --- | --- | --- | --- | --- | --- |
|  | **Questions** | | No | Don’t know | yes | **References**  (please give legal references) | **Comments**  (please tick box to indicate at which level each law is framed: N= National, R= regional, L=local) |
| 1 | Apart from the reporting duties imposed by IHR 2005 (article 6), do you have any other national sources of legal duty to report communicable diseases in your country? | |  |  |  |  | \| N \|  \| \| --- \| --- \| \| R \|  \| \| L \|  \| |
| 2 | Do you have laws which impose a duty to report cases of disease: | |  |  |  |  |  |
|  | a | At national level? |  |  |  |  |  |
|  | b | At regional level? |  |  |  |  |  |
|  | c | At local level? |  |  |  |  |  |
| 3 | Are the communicable diseases to be reported specified in a list? | |  |  |  |  |  |
| 4 | Is there a duty to report **any** disease which creates a *public health risk*? | |  |  |  |  |  |
| 5 | Is there a duty to report *human influenza*? (see glossary definition) | |  |  |  |  |  |
| 6 | Who has a legal duty to report cases of disease? | | | | |  |  |

|  | **Questions** | No | Don’t know | yes | **References**  (please give legal references) | **Comments**  (please tick box to indicate at which level each law is framed: N= National, R= regional, L=local) |
| --- | --- | --- | --- | --- | --- | --- |
| 7 | To whom must the disease be reported? | |  |  | |  |
| 8 | Can natural persons with a reporting duty be *sanctioned* for non-compliance? |  |  |  |  |  |
| 9 | Are there any other reporting obligation resulting from agreement with other states (Schengen, EFTA, Nordic Agreement, etc)? |  |  |  |  |  |

|  | **Questions** | | | No | Don’t know | yes | **References**  (please give legal references) | **Comments**  (please tick box to indicate at which level each law is framed: N= National, R= regional, L=local) |
| --- | --- | --- | --- | --- | --- | --- | --- | --- |
| 10 | Do your laws authorise reporting of *personal identification information* in relation to individual cases? | | |  |  |  |  | \| N \|  \| \| --- \| --- \| \| R \|  \| \| L \|  \| |
| 11 | Will the persons who are the subject of the reporting duty: | | |  |  |  |  |  |
|  | a | | be informed that this information is being reported? |  |  |  |  |  |
|  | b | | have the right of access to the information provided? |  |  |  |  |  |
| 12 | Do your laws require measures to be taken to protect the privacy of the persons who are subject to reporting? | | |  |  |  |  |  |
|  | a | | What measures? | | | |  |  |
| 13 | Do your laws recognise any circumstances in which the public health need for health information overrides the right of the individual to privacy? | | |  |  |  |  |  |
|  | a | Please give details | | | | |  |  |

| **Other issues** | | | | | | | | |
| --- | --- | --- | --- | --- | --- | --- | --- | --- |
|  | **Questions** | | | No | Don’t know | yes | **References**  (please give legal references) | **Comments**  (please tick box to indicate at which level each law is framed: N= National, R= regional, L=local) |
| 14 | Do your laws authorise *biological samples* to be taken from patients without *consent*? | | |  |  |  |  | \| N \|  \| \| --- \| --- \| \| R \|  \| \| L \|  \| |
| 15 | Do your laws require *exposed/infected* persons: | | |  |  |  |  | \| N \|  \| \| --- \| --- \| \| R \|  \| \| L \|  \| |
|  | a | | to assist with *source* and *contact tracing* by public health officers? |  |  |  |  |  |
|  | b | | to answer questions about their health or other circumstances? |  |  |  |  |  |
|  | c | | to answer questions about the health or circumstances of another person? |  |  |  |  |  |
| 16 | Will such persons, including persons subject to contact tracing, have the right of access to information provided? | | |  |  |  |  |  |
| 17 | Do your laws require measures to be taken to protect the privacy of such persons? | | |  |  |  |  |  |
|  | a | What measures? | | | | |  |  |
| 18 | Do your laws authorise a *compulsory* order that a person attend training or counselling  sessions on how to reduce the risk of infecting or contaminating others? | | |  |  |  |  | \| N \|  \| \| --- \| --- \| \| R \|  \| \| L \|  \| |

| **Compulsory measures in relation to persons within your country** | | | | | | | |
| --- | --- | --- | --- | --- | --- | --- | --- |
| **Screening and medical examination** | | | | | | | |
|  | **Questions** | | No | Don’t know | yes | **References**  (please give legal references) | **Comments**  (please tick box to indicate at which level each law is framed: N= National, R= regional, L=local) |
| 19 | Do your laws authorise compulsory *population screening* for communicable disease? | |  |  |  |  | \| N \|  \| \| --- \| --- \| \| R \|  \| \| L \|  \| |
|  | a | Who has the power to carry out *population screening*? |  | | | |  |
| 20 | Do your laws authorise compulsory *non-invasive medical examination*? | |  |  |  |  | \| N \|  \| \| --- \| --- \| \| R \|  \| \| L \|  \| |
|  | a | On what grounds can compulsory *non-invasive* *medical examination* be ordered? | | | |  |  |
|  | b | Who has the power to carry out compulsory *non-invasive* *medical examination*? | | | |  |  |

|  | **Questions** | | No | Don’t know | yes | **References**  (please give legal references) | **Comments**  (please tick box to indicate at which level each law is framed: N= National, R= regional, L=local) |
| --- | --- | --- | --- | --- | --- | --- | --- |
| 21 | Do your laws authorise compulsory *invasive* medical examination? | |  |  |  |  | \| N \|  \| \| --- \| --- \| \| R \|  \| \| L \|  \| |
|  | a | On what grounds? |  | | | |  |
|  | b | Who has power to carry out compulsory *invasive* medical examination? |  | | | |  |

| **Isolation and quarantine** | | | | | | | |
| --- | --- | --- | --- | --- | --- | --- | --- |
|  | **Questions** | | No | Don’t know | yes | **References**  (please give legal references) | **Comments**  (please tick box to indicate at which level each law is framed: N= National, R= regional, L=local) |
| 22 | Do your country’s laws authorise compulsory *isolation* of persons with symptoms of influenza? | |  |  |  |  | \| N \|  \| \| --- \| --- \| \| R \|  \| \| L \|  \| |
|  | a | Does an isolated person have *a right of appea*l against isolation? |  |  |  |  |  |
|  | b | Does an isolated person have any other means of challenging isolation? |  |  |  |  |  |
|  |  | If yes, what means? | | | |  |  |

|  | **Questions** | | No | Don’t know | yes | **References**  (please give legal references) | **Comments**  (please tick box to indicate at which level each law is framed: N= National, R= regional, L=local) |
| --- | --- | --- | --- | --- | --- | --- | --- |
| 23 | Where is isolation authorised to take place? | |  | | | | \| N \|  \| \| --- \| --- \| \| R \|  \| \| L \|  \| |
|  | a | At home? |  |  |  |  |  |
|  | b | In hospital? |  |  |  |  |  |
|  | c | In prison? |  |  |  |  |  |
|  | d | In a detention centre? |  |  |  |  |  |
|  | e | In designated premises? |  |  |  |  |  |
|  | F | Other? Give details |  |  |  |  |  |
| 24 | Do your laws limit how long a person can be isolated? | |  |  |  |  | \| N \|  \| \| --- \| --- \| \| R \|  \| \| L \|  \| |
|  | a | Until the person is no longer infectious? |  |  |  |  |  |
|  | b | Until the person ceases to pose a risk to public health? |  |  |  |  |  |
|  | c | Until the person is no longer infected? |  |  |  |  |  |
|  | d | For a set period of time? |  |  |  |  |  |
|  | e | Other? Give details |  |  |  |  |  |

|  | **Questions** | | No | Don’t know | yes | **References**  (please give legal references) | **Comments**  (please tick box to indicate at which level each law is framed: N= National, R= regional, L=local) |
| --- | --- | --- | --- | --- | --- | --- | --- |
| 25 | Do your laws require isolation to be reviewed after a period of time? | |  |  |  |  | \| N \|  \| \| --- \| --- \| \| R \|  \| \| L \|  \| |
|  | a | If yes, after how long? | | | |  |  |
| **Compensation** | | | | | | | |
|  | **Questions** | | No | Don’t know | yes | **References**  (please give legal references) | **Comments**  (please tick box to indicate at which level each law is framed: N= National, R= regional, L=local) |
| 26 | a | Do your laws **authorise** payment of compensation to persons who voluntarily agree to isolation? |  |  |  |  | \| N \|  \| \| --- \| --- \| \| R \|  \| \| L \|  \| |
|  | b | Do your laws **authorise** payment of compensation to persons who are compulsorily isolated? |  |  |  |  | \| N \|  \| \| --- \| --- \| \| R \|  \| \| L \|  \| |
|  | c | Do your laws **require** payment of compensation to persons isolated on disease grounds? |  |  |  |  | \| N \|  \| \| --- \| --- \| \| R \|  \| \| L \|  \| |
| 27 | Do your laws authorise *compulsory quarantine* of *contacts* of a person infected with influenza? | |  |  |  |  | \| N \|  \| \| --- \| --- \| \| R \|  \| \| L \|  \| |

|  | **Questions** | | No | Don’t know | yes | **References**  (please give legal references) | **Comments**  (please tick box to indicate at which level each law is framed: N= National, R= regional, L=local) |
| --- | --- | --- | --- | --- | --- | --- | --- |
| 28 | Where is quarantine authorised to take place? | | |  | | | \| N \|  \| \| --- \| --- \| \| R \|  \| \| L \|  \| |
|  | a | At home? |  |  |  |  |  |
|  | b | In hospital? |  |  |  |  |  |
|  | c | In prison? |  |  |  |  |  |
|  | d | In a detention centre? |  |  |  |  |  |
|  | e | In designated premises? |  |  |  |  |  |
|  | f | Other? Give details |  |  |  |  |  |
| 29 | Do your laws limit how long a person can be compulsorily quarantined? | |  |  |  |  | \| N \|  \| \| --- \| --- \| \| R \|  \| \| L \|  \| |
|  | a | Until it can be established that the person is not infected? |  |  |  |  |  |
|  | b | For a set period of time? |  |  |  |  |  |
|  | d | Other? Give details |  |  |  |  |  |
| 30 | Do your laws require quarantine to be reviewed after a period of time? | |  |  |  |  | \| N \|  \| \| --- \| --- \| \| R \|  \| \| L \|  \| |
|  | a | If yes, after how long? |  |  |  |  |  |

| **Compensation** | | | | | | | |
| --- | --- | --- | --- | --- | --- | --- | --- |
|  | **Questions** | | No | Don’t know | Yes | **References**  (please give legal references) | **Comments**  (please tick box to indicate at which level each law is framed: N= National, R= regional, L=local) |
| 31 | a | Do your laws **authorise** payment of compensation to persons who voluntarily agree to quarantine? |  |  |  |  | \| N \|  \| \| --- \| --- \| \| R \|  \| \| L \|  \| |
|  | b | Do your laws **authorise** payment of compensation to persons who are compulsorily quarantined? |  |  |  |  | \| N \|  \| \| --- \| --- \| \| R \|  \| \| L \|  \| |
|  | c | Do your laws **require** payment of compensation to persons quarantined on disease grounds? |  |  |  |  | \| N \|  \| \| --- \| --- \| \| R \|  \| \| L \|  \| |

| **Vaccination and prophylaxis** | | | | | | | |
| --- | --- | --- | --- | --- | --- | --- | --- |
|  | **Questions** | | No | Don’t know | yes | **References**  (please give legal references) | **Comments**  (please tick box to indicate at which level each law is framed: N= National, R= regional, L=local) |
| 32 | Do your laws authorise compulsory *vaccination* against influenza? | |  |  |  |  | \| N \|  \| \| --- \| --- \| \| R \|  \| \| L \|  \| |
|  | a | Are there any grounds for exemption from compulsory vaccination (e.g. religious, health grounds)? Give details. | | |  | |  |
| 33 | Do your laws authorise compulsory *prophylaxis* of potentially infected persons? | |  |  |  |  | \| N \|  \| \| --- \| --- \| \| R \|  \| \| L \|  \| |
|  | a | Of adults? |  | | | |  |
|  | b | Of children? |  | | | |  |
|  | c | Are there any grounds for exemption from compulsory *prophylaxis?* Give details. |  | | | |  |

| **Treatment and decontamination** | | | | | | | |
| --- | --- | --- | --- | --- | --- | --- | --- |
|  | **Questions** | | No | Don’t know | yes | **References**  (please give legal references) | **Comments**  (please tick box to indicate at which level each law is framed: N= National, R= regional, L=local) |
| 34 | Do your laws authorise compulsory *medical* treatment of infected persons? | |  |  |  |  | \| N \|  \| \| --- \| --- \| \| R \|  \| \| L \|  \| |
|  | a | Will compulsory *medical* treatment be paid for by the state? |  | | | |  |
|  | b | Will compulsory *medical* treatment be paid for by the body that authorises it? |  | | | |  |
|  | c | Will compulsory *medical* treatment be paid for by any other body/person? |  | | | |  |
|  | d | Are there any grounds for exemption from compulsory treatment? Give details. |  | | | |  |

|  | **Questions** | | No | Don’t know | yes | **References**  (please give legal references) | **Comments**  (please tick box to indicate at which level each law is framed: N= National, R= regional, L=local) |
| --- | --- | --- | --- | --- | --- | --- | --- |
| 35 | Do your laws authorise compulsory *disinfection* or *decontamination* of persons? | |  |  |  |  | \| N \|  \| \| --- \| --- \| \| R \|  \| \| L \|  \| |
|  | a | Are there any grounds for exemption from compulsory disinfection or decontamination of persons? Give details. |  | | | |  |
| **Restrictions** | | | | | | | |
|  | **Questions** | | No | Don’t know | yes | **References**  (please give legal references) | **Comments**  (please tick box to indicate at which level each law is framed: N= National, R= regional, L=local) |
| 36 | Do your laws authorise restriction of contact with other persons on grounds of communicable disease? | |  |  |  |  | \| N \|  \| \| --- \| --- \| \| R \|  \| \| L \|  \| |

| **General questions on compulsory powers** | | | | | | | |
| --- | --- | --- | --- | --- | --- | --- | --- |
|  | **Questions** | | No | Don’t know | yes | **References**  (please give legal references) | **Comments**  (please tick box to indicate at which level each law is framed: N= National, R= regional, L=local) |
| 37 | Do your laws authorise the exercise of compulsory powers on groups of persons as well as on individuals? | |  |  |  |  | \| N \|  \| \| --- \| --- \| \| R \|  \| \| L \|  \| |
| 38 | Is court authorisation needed for the exercise of compulsory powers? | |  |  |  |  | \| N \|  \| \| --- \| --- \| \| R \|  \| \| L \|  \| |
| 39 | Do persons who are the subject of a compulsory power have a *right of appeal*? | |  |  |  |  | \| N \|  \| \| --- \| --- \| \| R \|  \| \| L \|  \| |
|  | a | Do your laws provide legal assistance to people appealing? |  |  |  |  |  |
|  | b | Do persons who are the subject of compulsory powers have any other means of challenging them? |  |  |  |  |  |
|  | c | What means? | | | |  |  |
|  | d | Are there any limitations on the circumstances in which a compulsory power order can be used? Give details. | | | |  |  |

| **Criminal offences** | | | | | | | |
| --- | --- | --- | --- | --- | --- | --- | --- |
|  | **Questions** | | No | Don’t know | yes | **References**  (please give legal references) | **Comments**  (please tick box to indicate at which level each law is framed: N= National, R= regional, L=local) |
| 40 | In your country, is it an *offence* for a person: | |  |  |  |  |  |
|  | a | Not to comply with a compulsory power order? |  |  |  |  | \| N \|  \| \| --- \| --- \| \| R \|  \| \| L \|  \| |
|  | b | To **knowingly** expose another to risk of disease? |  |  |  |  |  |
|  | c | To **recklessly** expose another to risk of disease? |  |  |  |  |  |
| **Compulsory measures in relation persons in transit** | | | | | | | |
|  | **Questions** | | No | Don’t know | yes | **References**  (please give legal references) | **Comments**  (please tick box to indicate at which level each law is framed: N= National, R= regional, L=local) |
| 41 | Do your laws authorise compulsory screening of people *in transit* through your country? | |  |  |  |  | \| N \|  \| \| --- \| --- \| \| R \|  \| \| L \|  \| |
| 42 | Do your laws authorise compulsory *non invasive* medical examination of persons *in transit* through your country? | |  |  |  |  | \| N \|  \| \| --- \| --- \| \| R \|  \| \| L \|  \| |
| 43 | Do your laws authorise compulsory *medical treatment* of infected persons *in transit* through your country? | |  |  |  |  | \| N \|  \| \| --- \| --- \| \| R \|  \| \| L \|  \| |

| **Obligation to provide healthcare to people in transit** | | | | | | | |
| --- | --- | --- | --- | --- | --- | --- | --- |
|  | **Questions** | | No | Don’t know | yes | **References**  (please give legal references) | **Comments**  (please tick box to indicate at which level each law is framed: N= National, R= regional, L=local) |
| 44 | Is there a legal obligation to provide *healthcare* for infected persons *in transit?* | |  |  |  |  | \| N \|  \| \| --- \| --- \| \| R \|  \| \| L \|  \| |
|  | a | Who has the obligation to provide this healthcare? Give details | | | |  |  |
|  | b | Will healthcare for infected persons in transit be paid for by the state? |  |  |  |  |  |
|  | c | Will healthcare for infected persons in transit be paid for by the body/agency that authorises it? |  |  |  |  |  |
|  | d | Will healthcare for infected persons in transit be paid for by any other body/person? | | | |  |  |

| **Obligations of conveyance operators and airport authorities** | | | | | | | |
| --- | --- | --- | --- | --- | --- | --- | --- |
|  | **Questions** | | No | Don’t know | yes | **References**  (please give legal references) | **Comments**  (please tick box to indicate at which level each law is framed: N= National, R= regional, L=local) |
| 45 | Are *conveyance operators* or *crew* required by your laws to provide *personal data* of *travellers* to/from an *affected area*? | |  |  |  |  | \| N \|  \| \| --- \| --- \| \| R \|  \| \| L \|  \| |
| 46 | Do *conveyance operators* or *crew* have a duty to report disease? | |  |  |  |  | \| N \|  \| \| --- \| --- \| \| R \|  \| \| L \|  \| |
|  | a | If yes, can they be sanctioned for non-compliance? |  |  |  |  |  |
| 47 | Is there a legal obligation on airport authorities to provide health services to infected passengers? | |  |  |  |  | \| N \|  \| \| --- \| --- \| \| R \|  \| \| L \|  \| |

| **Border closure** | | | | | | | |
| --- | --- | --- | --- | --- | --- | --- | --- |
|  | **Questions** | | No | Don’t know | yes | **References**  (please give legal references) | **Comments**  (please tick box to indicate at which level each law is framed: N= National, R= regional, L=local) |
| 48 | In the event of the threat of a pandemic, do your laws authorise closure of your country’s *borders*? | |  |  |  |  | \| N \|  \| \| --- \| --- \| \| R \|  \| \| L \|  \| |
| 49 | If yes, do your laws authorise border closure to exclude citizens from: | |  |  |  |  | \| N \|  \| \| --- \| --- \| \| R \|  \| \| L \|  \| |
|  | a | European countries not member of the EU? |  |  |  |  |  |
|  | b | EU States? |  |  |  |  |  |
|  | c | States within the Schengen agreement? |  |  |  |  |  |
|  | d | EFTA States? |  |  |  |  |  |
|  | e | Switzerland? |  |  |  |  |  |
|  | f | Non-European countries? |  |  |  |  |  |
|  | g | Other? Give details |  |  |  |  |  |
| 50 | In case of border closure do your laws authorise transport of medical supplies through your country? | |  |  |  |  | \| N \|  \| \| --- \| --- \| \| R \|  \| \| L \|  \| |

| **Repatriation** | | | | | | | |
| --- | --- | --- | --- | --- | --- | --- | --- |
|  | **Questions** | | No | Don’t know | yes | **References**  (please give legal references) | **Comments**  (please tick box to indicate at which level each law is framed: N= National, R= regional, L=local) |
| 51 | If your borders are closed, do your laws authorise entry to be denied to your country’s  citizens returning from abroad? | |  |  |  |  | \| N \|  \| \| --- \| --- \| \| R \|  \| \| L \|  \| |
| 52 | Do your laws require your country to provide *repatriation services* to a citizen of another country who is in your country? | |  |  |  |  | \| N \|  \| \| --- \| --- \| \| R \|  \| \| L \|  \| |
|  | a | If not, do your laws require *assistance* to be provided to that person? |  |  |  |  |  |
|  | b | If yes, what type of assistance? | | | |  |  |

# V- narrative Part B - (pandemic period: who Phase 6)

**Influenza pandemic in europe**

**15 May 2009**

Widespread and uncontrolled human-to-human transmission is now occurring throughout S.E. Asia and the WHO announces that the pandemic alert level is being raised to **Phase 6**. This means that the world is now dealing with a **pandemic**.

In Europe, 20 countries are currently facing major HXNY influenza outbreaks. In your country, personal protective equipment and AV drugs are becoming scarce as suppliers are not able to keep up with the demand. These resource shortages pose issues of resource allocation. In accordance with its pandemic preparedness plan, your country prioritizes the distribution of personal protective equipment and of AV prophylaxis to healthcare workers working in healthcare facilities.

In an attempt to control disease spread, additional social distancing measures are being implemented by a number of European countries. In your country, internal travel restrictions are put into force in order to protect the south of the country, which is less affected by the pandemic than the north. All kindergartens and schools (all educational levels) are closed, and mass social events are prohibited. Visiting hours in healthcare facilities are reduced and your country envisages suspending all visiting rights. For reasons of hygiene, the authorities in one of the administrative regions of your countries is considering cremating patients who have died who were suspected or confirmed to be infected, regardless of the burial wishes of families and of religious considerations.

In your country, demand for healthcare largely exceeds capacity. Many healthcare facilities are overcrowded and need to devote the bulk of their resources to pandemic management. This situation severely compromises the delivery of other essential healthcare services, such as surgical and obstetric services. In order to increase capacity, private and public buildings, furniture and vehicles are requisitioned for isolation of infected persons and for use as makeshift hospitals. Pressure is growing on healthcare staff and some refuse to attend work. In order to maintain sufficient staff around the clock, hospital managers request that staff work longer shifts and additional days. Some managers are looking to alternative sources of nursing staff. There are talks of recruiting retired nurses and 3^rd^ year nursing students, and of requisitioning veterinary and dental nurses.

A large hospital in which infection control protocols have been breached, is isolated, and all staff and patients are confined to the hospital. The crisis faced by the hospital is covered in media news broadcasts. The images show patients dying as a result of poor health care. Your country feels that this situation might aggravate the tension in the population, and places an embargo on any news reporting which might potentially provoke public panic.

**1 June 2009**

The media announces that your country is amongst the first few European countries that will soon receive a delivery of the licensed HXNY influenza vaccine. The vaccine stocks should be sufficient to cover 5% of your country’s population. This news raises not only issues of vaccine distribution and prioritization but also sparks concerns of vaccination compliance by those persons identified as most needing to be vaccinated. Your country fears an influx of people from other European countries trying to cross the borders in the hope of accessing the vaccine.

**15 June 2009**

All countries in the European region are now affected by the pandemic, albeit to varying degrees. Economic activities are now, in most countries, reduced to a minimum and workers who have been exposed to persons with influenza are voluntarily quarantined at home. Concerns about the maintenance of adequate staffing levels in vital services are rising as high rates of absenteeism threaten the continuity of their business operations. For example, in one city, employees of the water services refuse to attend work, fearing they will be exposed to the disease if they leave their homes. The water company is understaffed to the point that it cannot continue to operate. This compromises the delivery of safe water supplies to hundreds of thousands of people. Also, there is a major outbreak of influenza in one of the main prisons of the country and the majority of prison guards are no longer prepared to work, claiming that the situation is unsafe.

Your country plans to requisition vaccine production from its national vaccine providers so that your country’s needs can be met.

**END OF NARRATIVE PART B**

# VI- Legal questions - part B: Pandemic period

**Instructions for completing the pandemic stage questionnaire (part B)**

Some public health powers in a pandemic might derive from Emergency powers legislation or laws rather than from public health legislation or laws.

In the **Comments** column, please indicate, by ticking the appropriate box, whether the relevant law is contained in emergency powers laws/legislation or within public health law/legislation: box **E** for *Emergency power law/legislation*, and box **H** for *Health* or *Public Health law*/*legislation*. If laws are contained in both, please tick both boxes, and where further explanation is required, please comment in the **Comments** column.

|  | **Obligation to provide health care** | | | | | | |
| --- | --- | --- | --- | --- | --- | --- | --- |
|  | **Questions** | | No | Don’t know | yes | **References**  (please give legal references) | **Comments**  (Please tick box: E= Emergency power; H= Public Health or Health Law) |
| 1 | In case of a pandemic, is there a legal duty in your country to provide healthcare for the following infected persons: | |  |  |  |  | \| E \|  \| \| --- \| --- \| \| H \|  \| |
|  | a | nationals of a European state not a member of the EU who are *resident* in your country? |  |  |  |  |  |
|  | b | EU nationals who are resident in your country? |  |  |  |  |  |
|  | c | nationals of a Schengen Member State who are resident in your country? |  |  |  |  |  |
|  | d | EFTA national citizens who are resident in your country? |  |  |  |  |  |
|  | e | citizens of Switzerland who are in resident your country? |  |  |  |  |  |
|  | f | non-European nationals who are resident in your country? |  |  |  |  |  |
|  | g | *visitors* to your country who are citizens of an European country which is not a member of the EU? |  |  |  |  |  |
|  | h | visitors to your country from EU countries? |  |  |  |  |  |
|  | i | visitors to your country from Schengen member states? |  |  |  |  |  |
|  | j | visitors to your country from Switzerland? |  |  |  |  |  |
|  | k | visitors to your country from an EFTA state? |  |  |  |  |  |
|  | l | visitors to your country from a country outside Europe? |  |  |  |  |  |

|  |  | **Questions 1 (continued)** | No | Don’t know | yes | **References**  (please give legal references) | **Comments**  (Please tick box: E= Emergency power; H= Public Health or Health Law) |
| --- | --- | --- | --- | --- | --- | --- | --- |
|  | m | persons without *health insurance*? |  |  |  |  |  |
|  | n | *undocumented immigrants?* |  |  |  |  |  |
|  | o | *refugees?* |  |  |  |  |  |

| **Prioritisation of healthcare** | | | | | | | | |
| --- | --- | --- | --- | --- | --- | --- | --- | --- |
|  | **Questions** | | | No | Don’t know | yes | **References**  (please give legal references) | **Comments**  (Please tick box: E= Emergency power; H= Public Health or Health Law) |
| 2 | In the event of a pandemic, do your laws address provision of healthcare for patients *other* than those infected by influenza? | | |  |  |  |  | \| E \|  \| \| --- \| --- \| \| H \|  \| |
|  | a | | If yes, is there a legal obligation in a pandemic stage to continue to provide healthcare for persons other than those infected by influenza? |  |  |  |  |  |
| 3 | Do your laws authorise prioritization of patients in access to healthcare in a pandemic stage? | | |  |  |  |  | \| E \|  \| \| --- \| --- \| \| H \|  \| |
| 4 | Do your laws authorise prioritisation to particular groups of persons in relation to access: | | |  |  |  |  | \| E \|  \| \| --- \| --- \| \| H \|  \| |
|  | a | to antivirals? | |  |  |  |  |  |
|  | b | to vaccination? | |  |  |  |  |  |
|  | c | If yes, who has priority? Give details | | | | |  |  |

| **Personal protective equipment** | | | | | | |
| --- | --- | --- | --- | --- | --- | --- |
|  | **Questions** | No | Don’t know | yes | **References**  (please give legal references) | **Comments**  (Please tick box: E= Emergency power; H= Public Health or Health Law) |
| 5 | Do your laws authorise the compulsory wearing of disease protective equipment in public places? |  |  |  |  | \| E \|  \| \| --- \| --- \| \| H \|  \| |
| 6 | Do your laws authorise provision of disease protective equipment free of charge? |  |  |  |  | \| E \|  \| \| --- \| --- \| \| H \|  \| |
| **Distancing measures** | | | | | | |
|  | **Questions** | No | Don’t know | yes | **References**  (please give legal references) | **Comments**  (Please tick box: E= Emergency power; H= Public Health or Health Law) |
| 7 | Do your laws authorise prohibition of *mass gatherings* on public health grounds? |  |  |  |  | \| E \|  \| \| --- \| --- \| \| H \|  \| |
| 8 | Do your laws authorise hospitals to refuse access to visitors to patients of the hospital? |  |  |  |  | \| E \|  \| \| --- \| --- \| \| H \|  \| |

| **Closure, isolation and evacuation of facilities** | | | | | | | | |
| --- | --- | --- | --- | --- | --- | --- | --- | --- |
|  | **Questions** | | | No | Don’t know | yes | **References**  (please give legal references) | **Comments**  (Please tick box: E= Emergency power; H= Public Health or Health Law) |
| 9 | Do your laws provide for closure of: | | |  |  |  |  | \| E \|  \| \| --- \| --- \| \| H \|  \| |
|  | a | | private facilities? (private clubs, restaurants etc.) |  |  |  |  |  |
|  | b | | public premises (parks, museums etc)? |  |  |  |  |  |
|  | c | | hotels? |  |  |  |  |  |
|  | d | | leisure facilities? (cinemas, theatres, gyms etc) ? |  |  |  |  |  |
|  | e | | sports facilities? |  |  |  |  |  |
|  | f | | hospitals? |  |  |  |  |  |
|  | g | | kindergartens and schools? |  |  |  |  |  |
|  | h | | universities? |  |  |  |  |  |
|  | i | | prisons? |  |  |  |  |  |
|  | j | | public transport facilities? |  |  |  |  |  |
| 10 | Do your laws authorise the evacuation of premises on public health grounds? | | |  |  |  |  | \| E \|  \| \| --- \| --- \| \| H \|  \| |
| 11 | Do your laws authorise isolation of an institution/building/physical space? | | |  |  |  |  | \| E \|  \| \| --- \| --- \| \| H \|  \| |
|  | a | Does this power include isolation of a hospital? | |  |  |  |  |  |
| 12 | Do your laws require a child who is sick to abstain from attending school during pandemic? | | |  |  |  |  | \| E \|  \| \| --- \| --- \| \| H \|  \| |
| 13 | Do your laws authorise regulation of blood/organ donation in a pandemic? | | |  |  |  |  | \| E \|  \| \| --- \| --- \| \| H \|  \| |
|  | **Questions** | | | No | Don’t know | yes | **References**  (please give legal references) | **Comments**  (Please tick box: E= Emergency power; H= Public Health or Health Law) |
| 14 | Is a court order required to authorise any of these measures?  Give details | | |  |  |  |  | \| E \|  \| \| --- \| --- \| \| H \|  \| |
| **Restriction of movement** | | | | | | | | |
|  | **Questions** | | | No | Don’t know | yes | **References**  (please give legal references) | **Comments**  (Please tick box: E= Emergency power; H= Public Health or Health Law) |
| 15 | Do your laws authorise restriction of travel to/from local affected areas within your country in a pandemic? | | |  |  |  |  | \| E \|  \| \| --- \| --- \| \| H \|  \| |
| 16 | Do your laws allow for establishment of a *cordon sanitaire*? | | |  |  |  |  | \| E \|  \| \| --- \| --- \| \| H \|  \| |

| **Vaccines** | | | | | | | |
| --- | --- | --- | --- | --- | --- | --- | --- |
|  | **Questions** | | No | Don’t know | yes | **References**  (please give legal references) | **Comments**  (Please tick box: E= Emergency power; H= Public Health or Health Law) |
| 17 | Do your laws authorise the monitoring of *vaccine coverage* of the population during a pandemic? | |  |  |  |  | \| E \|  \| \| --- \| --- \| \| H \|  \| |
| 18 | Do your laws require reporting of the *adverse effects* of vaccination? | |  |  |  |  | \| E \|  \| \| --- \| --- \| \| H \|  \| |
|  | a | Who has the duty to report? |  | | | |  |
|  | b | Can professionals with a reporting duty be sanctioned for not reporting? |  |  |  |  |  |

|  | **Questions** | | | | No | Don’t know | | yes | **References**  (please give legal references) | **Comments**  (Please tick box: E= Emergency power; H= Public Health or Health Law) |
| --- | --- | --- | --- | --- | --- | --- | --- | --- | --- | --- |
| 19 | Do your laws authorise *compulsory* mass vaccination programmes? | | | |  |  | |  |  | \| E \|  \| \| --- \| --- \| \| H \|  \| |
|  | a | | | Do your laws provide for *compulsory* vaccination as part of a vaccination programme to be free of cost to the person vaccinated? |  |  | |  |  |  |
|  | b | | | Are there any grounds for exemption from compulsory mass vaccination? Give details. | | |  | | |  |
| 20 | Do your laws authorise the administration of *unlicensed vaccines* in a pandemic? | | | |  |  | |  |  | \| E \|  \| \| --- \| --- \| \| H \|  \| |
| 21 | If yes, do your laws provide that persons who are subject to the administration of unlicensed vaccines be informed of possible risks? | | | |  |  | |  |  |  |
| 22 | Do your laws provide for compensation for any damage resulting from administration of unlicensed vaccines? | | | |  |  | |  |  |  |
|  | a | | Will compensation be paid for by the state? | |  |  | |  |  |  |
|  | b | | Will compensation be paid for by the body that authorises the administration of the unlicensed vaccines? | |  |  | |  |  |  |
|  | c | | Will compensation be paid for by any other body/person? | |  |  | |  |  |  |
| 23 | Do your laws authorise the administration of *unlicensed prophylaxis* in a pandemic? | | | |  |  | |  |  | \| E \|  \| \| --- \| --- \| \| H \|  \| |
| 24 | If yes, do your laws provide that persons who are subject to the administration of unlicensed *prophylaxis* be informed of possible risks? | | | |  |  | |  |  |  |
| 25 | Do your laws provide for compensation for any damage resulting from administration of unlicensed *prophylaxis*? | | | |  |  | |  |  |  |
|  | a | Will compensation be paid for by the state? | | |  |  | |  |  |  |
|  | b | Will compensation be paid for by the body that authorises the administration of the unlicensed *prophylaxis*? | | |  |  | |  |  |  |
|  | c | Will compensation be paid for by any other body/person? | | |  |  | |  |  |  |

| **Requisition of persons** | | | | | | | |
| --- | --- | --- | --- | --- | --- | --- | --- |
|  | **Questions** | | No | Don’t know | yes | **References**  (please give legal references) | **Comments**  (Please tick box: E= Emergency power; H= Public Health or Health Law) |
| 26 | In a pandemic, do your laws authorise *requisition* of persons? | |  |  |  |  | \| E \|  \| \| --- \| --- \| \| H \|  \| |
|  | b | Do your laws **authorise** compensation to be paid for requisitioned persons? |  |  |  |  |  |
|  | c | Do your laws **require** compensation to be paid for requisitioned persons? |  |  |  |  |  |
| **Other staff issues** | | | | | | | |
|  | **Questions** | | No | Don’t know | yes | **References**  (please give legal references) | **Comments**  (Please tick box: E= Emergency power; H= Public Health or Health Law) |
| 27 | Do your laws provide a power to oblige workers to continue to work in a pandemic? | |  |  |  |  | \| E \|  \| \| --- \| --- \| \| H \|  \| |
| 28 | Do your laws allow an employee to refuse to work in circumstances which pose a risk to the employee’s health or safety? | |  |  |  |  | \| E \|  \| \| --- \| --- \| \| H \|  \| |
| 29 | Do your laws authorise unlicensed staff (e.g. medical students, retired doctors, nurses etc.)  to be required to perform medical acts in a pandemic? | |  |  |  |  | \| E \|  \| \| --- \| --- \| \| H \|  \| |
| 30 | Do your laws authorise medical staff to be redeployed to healthcare duties for which they are not trained in response to the needs of infected patients in a pandemic? (For example obstetricians, dentists etc. deployed to work with infected patients) | |  |  |  |  | \| E \|  \| \| --- \| --- \| \| H \|  \| |
| 31 | Do your laws authorise legal standards of treatment care to be suspended/reduced in a  pandemic? | |  |  |  |  | \| E \|  \| \| --- \| --- \| \| H \|  \| |

|  | **Questions** | | No | Don’t know | yes | **References**  (please give legal references) | **Comments**  (Please tick box: E= Emergency power; H= Public Health or Health Law) |
| --- | --- | --- | --- | --- | --- | --- | --- |
| 32 | Do your laws authorise healthcare staff to be required to work overtime in a pandemic? | |  |  |  |  | \| E \|  \| \| --- \| --- \| \| H \|  \| |
| **Requisition of premises** | | | | | | | |
|  | **Questions** | | No | Don’t know | yes | **References**  (please give legal references) | **Comments**  (Please tick box: E= Emergency power; H= Public Health or Health Law) |
| 33 | Do your laws authorise *requisition* of premises in a pandemic? | |  |  |  |  | \| E \|  \| \| --- \| --- \| \| H \|  \| |
| 34 | Do your laws authorise private hospitals to be requisitioned for the care of infected patients in a pandemic? | |  |  |  |  | \| E \|  \| \| --- \| --- \| \| H \|  \| |
| 35 | Do your laws authorise other private premises to be requisitioned for the purposes of isolation, quarantine or care of infected patients? | |  |  |  |  | \| E \|  \| \| --- \| --- \| \| H \|  \| |
|  | a | Do your laws **authorise** compensation to be paid for requisitioned property? |  |  |  |  |  |
|  | b | Do your laws **require** compensation to be paid for requisitioned property? |  |  |  |  |  |
| 36 | Do your laws authorise *compulsory* disinfection or decontamination of premises or structures as a disease control measure? | |  |  |  |  | \| E \|  \| \| --- \| --- \| \| H \|  \| |

| **Requisition of goods** | | | | | | |
| --- | --- | --- | --- | --- | --- | --- |
|  | **Questions** | No | Don’t know | yes | **References**  (please give legal references) | **Comments**  (Please tick box: E= Emergency power; H= Public Health or Health Law) |
| 37 | Do your laws authorise requisition of vehicles for emergency services in a pandemic? |  |  |  |  | \| E \|  \| \| --- \| --- \| \| H \|  \| |
| 38 | Do your laws authorise disinfection or decontamination of vehicles (e.g. ambulances) as a disease control measure? |  |  |  |  | \| E \|  \| \| --- \| --- \| \| H \|  \| |
| 39 | Do your laws authorise requisition of goods (such as medical equipment, drugs) in a pandemic? |  |  |  |  | \| E \|  \| \| --- \| --- \| \| H \|  \| |
| 40 | Do your laws authorise compulsory disinfection or decontamination of goods as a disease control measure? |  |  |  |  | \| E \|  \| \| --- \| --- \| \| H \|  \| |
| 41 | Do your laws authorise the seizure and detention of goods in a pandemic? |  |  |  |  | \| E \|  \| \| --- \| --- \| \| H \|  \| |
| 42 | Do your laws authorise compensation to be paid for requisitioned goods and vehicles? |  |  |  |  | \| E \|  \| \| --- \| --- \| \| H \|  \| |
| 43 | Do your laws authorise the overriding of *intellectual property rights* in relation to vaccines, antivirals or other necessary medical equipment in a pandemic? |  |  |  |  | \| E \|  \| \| --- \| --- \| \| H \|  \| |

| **Burial of deceased persons** | | | | | | | |
| --- | --- | --- | --- | --- | --- | --- | --- |
|  | **Questions** | | No | Don’t know | yes | **References**  (please give legal references) | **Comments**  (Please tick box: E= Emergency power; H= Public Health or Health Law) |
| 44 | Do your laws regulate the means of burial of deceased infected persons in a pandemic? | |  |  |  |  | \| E \|  \| \| --- \| --- \| \| H \|  \| |
|  | a | Are there any grounds for exemption from state regulation of means of burial? Give details. | | |  | |  |
| **Prisons** | | | | | | | |
|  | **Questions** | | No | Don’t know | yes | **References**  (please give legal references) | **Comments**  (Please tick box: E= Emergency power; H= Public Health or Health Law) |
| 45 | Do your laws make any provision in relation to the operation of prisons during a pandemic? If so, what provisions? | |  |  |  |  | \| E \|  \| \| --- \| --- \| \| H \|  \| |

| **Communication during a pandemic** | | | | | | |
| --- | --- | --- | --- | --- | --- | --- |
|  | **Questions** | No | Don’t know | yes | **References**  (please give legal references) | **Comments**  (Please tick box: E= Emergency power; H= Public Health or Health Law) |
| 46 | In the case of a pandemic, do your laws authorise censorship of information and/or control of the media? |  |  |  |  | \| E \|  \| \| --- \| --- \| \| H \|  \| |
| 47 | Who is authorized to communicate information to the public during a pandemic? | | | |  | \| E \|  \| \| --- \| --- \| \| H \|  \| |

# VIII- Glossary

## A

Adverse effects: harmful and undesired effects resulting from a drug, vaccine or medical treatment.

Affected: persons, baggage, cargo, containers, conveyances, goods, postal parcels or human remains that are infected or contaminated, or carry sources of infection or contamination, so as to constitute a public health risk. ^[[8]](#footnote-8)^

Affected area: a geographical location specifically for which health measures have been recommended by WHO under IHR 2005.^[[9]](#footnote-9)^

Airport authorities: see: ‘border guard’.

Antiviral (or AV): A drug that is used to prevent or cure a disease caused by a virus.^[[10]](#footnote-10)^

Assistance: any measures designed by a country to help its citizens in an affected country, or to help others countries’ nationals within its territory, in order to control or limit the spread of disease. For example provision of food, supplies, antivirals, vaccines and medications; assistance with repatriation, etc.

Authorise: to allow or empower designated persons/authorities to implement specific disease control measures which would otherwise contravene laws.

Avian influenza (also known as ‘bird flu’): An infection caused by an avian influenza virus, which primarily affects birds. There may be bird-to-bird transmission and occasional bird-to-human transmission. See also: ‘human influenza’; ‘pandemic influenza’; seasonal influenza’; ‘influenza’

## **B**

Biological sample: A biological specimen including, for example, blood, tissue, urine, etc. In the context of the PHLawFlu project, biological samples refer to samples taken from humans or animals suspected to be infected with influenza.^[[11]](#footnote-11)^

### Border:

In the context of the Schengen agreement:

*Internal borders^[[12]](#footnote-12)^***:**

(a) the common land borders, including river and lake borders, of the Member States;

(b) the airports of the Member States for internal flights;

(c) sea, river and lake ports of the Member States for regular ferry connections;

*External borders:*

- the EU Member States’ land borders, including river and lake borders, sea borders and their airports, river ports, sea ports and lake ports, provided that they are not internal borders.
- An initial entry point into EU.^[[13]](#footnote-13)^
- the external borders of the [Schengen agreement] Member States with third countries.^[[14]](#footnote-14)^

Border checks: the checks carried out at border crossing points, to ensure that persons, including their means of transport and the objects in their possession, may be authorised to enter the territory of the Member States or authorised to leave it.^[[15]](#footnote-15)^

Border closure: restriction of movement of people and goods to or from a country.

### Border control:

*In the context of the Schengen agreement:* the activity carried out at a border in response exclusively to an intention to cross or the act of crossing that border, regardless of any other consideration, consisting of border checks and border surveillance.^[[16]](#footnote-16)^ See also: ‘international travel and border controls’.

*International travel and border controls:* Measures designed to limit and/or con­trol the spread of infection across entry points to a country (by road, air, sea, etc). They can include travel advisories or restrictions, entry or exit screen­ing, reporting, health alert notices, collection and dissemination of passenger information, etc.^[[17]](#footnote-17)^

Border crossing point: any crossing-point authorised by the competent authorities for the crossing of external borders.^[[18]](#footnote-18)^

Border guard: any public official assigned, in accordance with national law, to a border crossing point or along the border or the immediate vicinity of that border who carries out, in accordance with law, border control tasks.^[[19]](#footnote-19)^

Border surveillance: the surveillance of borders between border crossing points and the surveillance of border crossing points outside the fixed opening hours, in order to prevent persons from circumventing border checks.^[[20]](#footnote-20)^

## **C**

Carrier: any natural or legal person whose occupation is to provide passenger transport by air.^[[21]](#footnote-21)^ Carrier as a medical term, see: ‘carrier (in medical terminology)’

Carrier (in medical terminology): an animal/person infected with an agent that causes a disease but shows no sign of illness. Asymptomatic carriers shed the causative agent, such as a virus or bacteria, and so can pass the disease on to others. ^[[22]](#footnote-22)^

Case: an individual infected by a pathogenic agent with or without clinical signs. ^[[23]](#footnote-23)^

Civil disaster: an event not originating from war or armed conflict and which causes considerable damage, injury or loss of life. An influenza pandemic might be considered a civil disaster.

Communicable disease: a disease that can be transmitted from one living being to another.^[[24]](#footnote-24)^

Compensation: in the context of the PHLawFlu project, a sum of money awarded to compensate for loss, injury or inconvenience resulting from measures to control communicable diseases or limit their spread (including measures such as isolation, quarantine, requisition, vaccination, etc.). See also: ‘project’.

Compulsory powers: powers provided by law to designated individuals/bodies to enable them to require persons to undertake disease control measures. For example powers of compulsory isolation, compulsory treatment or compulsory vaccination.

Consent: voluntary agreement to an action or treatment, on the basis of an understanding of the nature and consequences of the action or treatment.

*In the context of personal data***:** any freely given specific and informed indication of his/her wishes by which the data subject signifies his/her agreement to personal data relating to him being processed.^[[25]](#footnote-25)^

Contact: individuals exposed to someone with an infectious disease, including family members, roommates or housemates, close friends, co-workers, classmates, and others.^[[26]](#footnote-26)^

Contact tracing: [Identification](http://cancerweb.ncl.ac.uk/cgi-bin/omd?Identification) of [persons](http://cancerweb.ncl.ac.uk/cgi-bin/omd?persons) who have had contact with an [infected](http://cancerweb.ncl.ac.uk/cgi-bin/omd?infected) [person](http://cancerweb.ncl.ac.uk/cgi-bin/omd?person), animal or contaminated [environment](http://cancerweb.ncl.ac.uk/cgi-bin/omd?environment) where such contact might have provided the opportunity to acquire the [infection](http://cancerweb.ncl.ac.uk/cgi-bin/omd?infection).^[[27]](#footnote-27)^

Containment measures: measures designed to prevent the transmission of disease.

Contamination: the presence of an infectious or toxic agent or matter on a human or animal body surface, in or on a product prepared for consumption or on other inanimate objects, including conveyances, that may constitute a public health risk.^[[28]](#footnote-28)^

Conveyance: an aircraft, ship, train, road vehicle or other means of transport on an international voyage.^[[29]](#footnote-29)^

Conveyance operator: a natural or legal person in charge of a conveyance or their agent.^[[30]](#footnote-30)^

Cordon sanitaire (or sanitary cordon): a widespread, legally enforceable quarantine of a community or other large population group.^[[31]](#footnote-31)^

Court order: a writ issued by a court of law requiring a person to do something or to refrain from doing something.^[[32]](#footnote-32)^

Crew: persons on board a conveyance who are not passengers.^[[33]](#footnote-33)^

Crisis situation: a turning point in a disease; a crucial or decisive moment.

## **D**

Decontamination: a procedure whereby health measures are taken to eliminate an infectious or toxic agent or matter on a human or animal body surface, in or on a product prepared for consumption or on other inanimate objects, including conveyances, that may constitute a public health risk.^[[34]](#footnote-34)^

Delegated authority: an authority legally obtained from another superior body, in order to perform duties, make decisions or exercise powers. May also refer to the body exercising the delegated authority.

Detention: the act of detaining or the state of being detained, especially for medical purposes in order to limit the spread of a communicable disease.

DG SANCO: Health & Consumer Protection - Directorate General for Health and Consumers.^[[35]](#footnote-35)^

Disease: an illness or medical condition, irrespective of origin or source, that presents or could present significant harm to humans.^[[36]](#footnote-36)^

Disease cluster: a series of disease cases closely grouped in a specific geographical area or over a specific period of time. ^[[37]](#footnote-37)^

Disease outbreak: see: ‘epidemic’.

Disease protective equipment: see: ‘personal protective equipment’.

Disinfection: the procedure whereby health measures are taken to control or kill infectious agents on a human or animal body surface or in or on baggage, cargo, containers, conveyances, goods and postal parcels by direct exposure to chemical or physical agents.^[[38]](#footnote-38)^

Duty: a legal responsibility or obligation to do something.

## **E**

EC: European Commission.

ECDC: European Centre for Disease Prevention and Control (<http://ecdc.europa.eu/>).

EEA: European Economic Area. The EEA united from the 1 May 2004 the 25 EU Member States and the three EEA EFTA States (Iceland, Liechtenstein, and Norway) into an Internal Market governed by the same basic rules. These rules aim to enable [goods](http://secretariat.efta.int/Web/EuropeanEconomicArea/introduction/fourfreedoms/goods), [services](http://secretariat.efta.int/Web/EuropeanEconomicArea/introduction/fourfreedoms/FreeMovementOfServices), [capital](http://secretariat.efta.int/Web/EuropeanEconomicArea/introduction/fourfreedoms/FreeMovementOfCapital), and [persons](http://secretariat.efta.int/Web/EuropeanEconomicArea/introduction/fourfreedoms/FreeMovementOfPersons) to move freely about the EEA in an open and competitive environment, a concept referred to as the four freedoms. The objective of the EEA Agreement is to promote a continuous and balanced strengthening of trade and economic relations between the Contracting Parties with the view to creating a homogenous European Economic Area.^[[39]](#footnote-39)^

EFTA States: **The European Free Trade Association (EFTA)** is an intergovernmental organisation set up for the promotion of free trade and economic integration to the benefit of its four Member States: [Iceland](http://www.iceland.is/), [Liechtenstein](http://www.liechtenstein.li/en/), [Norway](http://www.norway.info/) and [Switzerland](http://www.swissworld.org/en/know/swiss_tags/?gclid=CNnb6PeW5JACFQ5aMAodf2OBOQ). It was founded in 1960 on the premise of free trade as a means of achieving growth and prosperity amongst its Member States as well as promoting closer economic co-operation between the Western European countries. The Association manages the [EFTA Convention](http://www.efta.int/content/legal-texts/efta-convention); EFTA’s worldwide network of [free trade and partnership agreements](http://www.efta.int/content/free-trade), and the [EEA Agreement](http://www.efta.int/content/eea/eea-agreement).^[[40]](#footnote-40)^

Emergency powers: Emergency powers address unexpected threats to authorise measures which would not normally be acceptable, or to provide powers as a last resort in the face of emergencies. They allow governments to make special temporary legislation (emergency regulations) where existing legislation is insufficient to respond in the most effective way. They are part of the Constitution in some countries, and can be found in separate legislation in others. Where the definition of an emergency includes a disease pandemic, such powers would allow public health authorities to take extraordinary measures where necessary to contain the pandemic.

Entering a country: persons crossing a country’s borders for purposes other than transit. See: ‘in transit’.

EPHLN: European Public Health Law Network ([www.ephln.org](http://www.ephln.org) ).

Epidemic: A disease occurring suddenly in humans in a community, region or country in numbers clearly in excess of normal. ^[[41]](#footnote-41)^

Epidemiological surveillance: the ongoing systematic collection, analysis, interpretation and dissemination of health data, essential to the planning, implementation, and evaluation of public health practice, closely integrated with the timely dissemination of these data to those who need to know in public health programs. ^[[42]](#footnote-42)^

Essential workers: In the context of the PHLawflu project, this refers to those workers who are essential for the continuity of business activities in any given organisation. See also: ‘vital workers’; ‘non-essential workers’; ‘project’.

EU: European Union.

EU Generic Preparedness documents: documents released by EU authorities on Influenza preparedness, such as:

- Communication from the Commission to the Council, the European Parliament, the European Economic and Social Committee of the Regions COM(2005) 605 final;
- Technical guidance document WD/C3 167.

EU states: see ‘Member States’.

European countries: **(**for the purposes of the PHLaw/Flu project): [Austria](http://europa.eu/abc/european_countries/eu_members/austria/index_en.htm), [Belgium](http://europa.eu/abc/european_countries/eu_members/belgium/index_en.htm), [Bulgaria](http://europa.eu/abc/european_countries/eu_members/bulgaria/index_en.htm), [Cyprus](http://europa.eu/abc/european_countries/eu_members/cyprus/index_en.htm), [Czech Republic](http://europa.eu/abc/european_countries/eu_members/czechrepublic/index_en.htm), [Denmark](http://europa.eu/abc/european_countries/eu_members/denmark/index_en.htm), [Estonia](http://europa.eu/abc/european_countries/eu_members/estonia/index_en.htm), [Finland](http://europa.eu/abc/european_countries/eu_members/finland/index_en.htm), [France](http://europa.eu/abc/european_countries/eu_members/france/index_en.htm), [Germany](http://europa.eu/abc/european_countries/eu_members/germany/index_en.htm), [Greece](http://europa.eu/abc/european_countries/eu_members/greece/index_en.htm), [Hungary](http://europa.eu/abc/european_countries/eu_members/hungary/index_en.htm), [Ireland](http://europa.eu/abc/european_countries/eu_members/ireland/index_en.htm), [Italy](http://europa.eu/abc/european_countries/eu_members/italy/index_en.htm), [Latvia](http://europa.eu/abc/european_countries/eu_members/latvia/index_en.htm), [Lithuania](http://europa.eu/abc/european_countries/eu_members/lithuania/index_en.htm), [Luxembourg](http://europa.eu/abc/european_countries/eu_members/luxembourg/index_en.htm), [Malta](http://europa.eu/abc/european_countries/eu_members/malta/index_en.htm), [Netherlands](http://europa.eu/abc/european_countries/eu_members/netherlands/index_en.htm), [Poland](http://europa.eu/abc/european_countries/eu_members/poland/index_en.htm), [Portugal](http://europa.eu/abc/european_countries/eu_members/portugal/index_en.htm), [Romania](http://europa.eu/abc/european_countries/eu_members/romania/index_en.htm), [Slovakia](http://europa.eu/abc/european_countries/eu_members/slovakia/index_en.htm), [Slovenia](http://europa.eu/abc/european_countries/eu_members/slovenia/index_en.htm), [Spain](http://europa.eu/abc/european_countries/eu_members/spain/index_en.htm), [Sweden](http://europa.eu/abc/european_countries/eu_members/sweden/index_en.htm), [United Kingdom](http://europa.eu/abc/european_countries/eu_members/unitedkingdom/index_en.htm). And: Croatia, Turkey, Iceland, Liechtenstein and Norway. See also: ‘project’.

Event: a manifestation of disease or an occurrence that creates a potential for disease.^[[43]](#footnote-43)^

Exposed: An individual who has spent time with or near someone who is infected by an infectious disease.

External borders: see ‘border’.

## **F**

Family member**^[[44]](#footnote-44)^ :** (of an EU citizen)

(a) the spouse;

(b) the partner with whom the Union citizen has contracted a registered partnership, on the basis of the legislation of a Member State, if the legislation of the host Member State treats registered partnerships as equivalent to marriage and in accordance with the conditions laid down in the relevant legislation of the host Member State;

(c) the direct descendants who are under the age of 21 or are dependants and those of the spouse or partner as defined in point (b);

(d) the dependent direct relatives in the ascending line and those of the spouse or partner as defined in point (b).

Federal (country): belonging or relating to a country consisting of a group of states independent in local matters but united under a central government for other purposes, such as defence, foreign policy. It can also refer to the central government of a group of federated states.^[[45]](#footnote-45)^

## **G**

Goods: tangible products, including animals and plants, transported on an international voyage, including for utilization on board a conveyance.^[[46]](#footnote-46)^

## **H**

Health care facility: an organization that employs health care workers and cares for patients/clients.^[[47]](#footnote-47)^ Includes any other facility /premises requisitioned during a pandemic for provision of health care.

Health care worker: Any person working in a health care facility, for example, medical officer, nurse, physiotherapist, cleaner, psychologist.^[[48]](#footnote-48)^ Includes any other person acquired by requisition or any other means to provide health care during a pandemic.

Health insurance: Insurance against [loss](http://www.investorwords.com/2896/loss.html) resulting from illness or bodily injury. [Health](http://www.businessdictionary.com/definition/health.html) [insurance](http://www.investorwords.com/2510/insurance.html) may provide [coverage](http://www.investorwords.com/1179/coverage.html) for medicines, [visits](http://www.businessdictionary.com/definition/visit.html) to the doctor or [emergency](http://www.businessdictionary.com/definition/emergency.html) room, hospital [stays](http://www.businessdictionary.com/definition/stay.html) and other medical [expenses](http://www.investorwords.com/1842/expense.html). [Policies](http://www.investorwords.com/3728/policy.html) differ in what they [cover](http://www.investorwords.com/1178/cover.html), the size of the [deductible](http://www.investorwords.com/1339/deductible.html) and/or co-payment, [limits](http://www.investorwords.com/2812/limit.html) of coverage and the [options](http://www.investorwords.com/3477/option.html) for treatment available to the [policyholder](http://www.investorwords.com/3729/policyholder.html).^[[49]](#footnote-49)^

Health measure: procedures applied to prevent the spread of disease or contamination; a health measure does not include law enforcement or security measures.^[[50]](#footnote-50)^

Health threat: a condition, agent or incident which may cause, directly or indirectly, ill health.^[[51]](#footnote-51)^

Host Member State: the Member State to which a Union citizen moves in order to exercise his/her right of free movement and residence.^[[52]](#footnote-52)^

Human influenza (also known as ‘flu’): an infection caused by a human influenza virus which affects and circulates in a sustainable manner in human populations. A contagious illness caused by a virus that attacks mainly the upper respiratory tract and usually lasts for about a week. The main characteristics of influenza are sudden onset of high fever, myalgia, headache and severe malaise, non-productive cough, sore throat, and rhinitis. Most people recover within one to two weeks without requiring any medical treatment. In the very young, the elderly and people suffering from specific medical conditions, influenza poses a serious risk and may lead to severe complications of underlying diseases, pneumonia and death. It can spread rapidly around the world in seasonal epidemics and hinder economic activities, as a result of hospital and other health costs and lost productivity.^[[53]](#footnote-53)^

Human-to-human: transmission of a disease from one human to another human.

HXNY: A fictitious subtype of influenza viruses for the purposes of the PHLawFlu study. See also: ‘influenza’; ‘human influenza’; ‘avian influenza’; ‘pandemic influenza’; ‘seasonal influenza’

## **I**

Ill person: an individual suffering from or affected with a physical ailment that may pose a public health risk.^[[54]](#footnote-54)^

Infection: the entry and development or multiplication of an infectious agent in the body of humans and animals that may constitute a public health risk.^[[55]](#footnote-55)^

Influenza: an infection caused by influenza viruses which can affect humans. Influenza viruses affect mainly the upper respiratory tract. The main characteristics of influenza are sudden onset of high fever, myalgia, headache and severe malaise, non-productive cough, sore throat, and rhinitis. See also: ‘human influenza’; ‘avian influenza’; ‘seasonal influenza’; ‘pandemic influenza’; ‘HXNY’.

Inoculation: see ‘vaccination’.

Inject (s) (ed): messages, or information about the next stage or events in a scenario such as the PHLawFlu pandemic disease scenario, are said to be injected when they are input into the exercise process for players to address. ^[[56]](#footnote-56)^

Intellectual property rights: Intellectual property refers to the creations of the human mind. Intellectual property rights protect the interests of creators by giving them property rights over their creations. Intellectual property relates to items of information or knowledge, which can be incorporated in tangible objects at the same time in an unlimited number of copies at different locations anywhere in the world. Intellectual property is usually divided into two branches, namely industrial property and copyright. Industrial property is more relevant to the PHLawFlu study, specifically in regard to vaccine and medicine production. The application of the term “industrial” is set out in the *Paris Convention for the Protection of Industrial Property* (Article 1 (3)): “Industrial property shall be understood in the broadest sense and shall apply not only to industry and commerce proper, but likewise to agricultural and extractive industries and to all manufactured or natural products, for example, wines, grain, tobacco leaf, fruit, cattle, minerals, mineral waters, beer, flowers, and flour.” Industrial property takes a range of forms, including patents to protect inventions; industrial designs, which are aesthetic creations determining the appearance of industrial products; trademarks, service marks, layout-designs of integrated circuits, commercial names and designations, as well as geographical indications, and protection against unfair competition.^[[57]](#footnote-57)^ See also: ‘project’.

Internal borders: see ‘border’.

Internal flight: (in the context of the Schengen agreement): any flight exclusively to or from the territories of the Schengen Member States and not landing in the territory of a third country.^[[58]](#footnote-58)^ Otherwise, any flight within a country.

International Health Regulations, IHR or Regulations: The International Health Regulations (IHR 2005) constitute an international legal instrument that is binding on 194 countries, including all countries that are the subject of the *PHLawFlu project*. The aim of the IHR is to assist the international community to prevent and respond to acute public health risks that have the potential to cross borders and threaten populations worldwide. The IHR may also apply to other public health emergencies such as chemical spills, leaks and dumping, or nuclear melt-downs. The IHR aim to limit interference with international traffic and trade while ensuring public health through the prevention of disease spread.

The IHR 2005, which entered into force on 15 June 2007, require countries to report certain disease outbreaks and public health events to WHO (see article 6). They define the rights and obligations of countries to report public health events, and establish a number of procedures that WHO must follow in its work to uphold global public health security. They also require countries to strengthen their existing capacities for public health surveillance and response.^[[59]](#footnote-59)^  States Parties have a time limit of five years maximum from the entry into force of the Regulations (15 June 2007) to meet the core capacity requirement. From 15 June 2007, State Parties have two years to assess their national structures and resources and develop national action plan; from 15 June 2009, they have three years to meet the core capacity requirements.^[[60]](#footnote-60)^

International voyage^[[61]](#footnote-61)^:

(a) in the case of a conveyance, a voyage between points of entry in the territories of more than one State, or a voyage between points of entry in the territory or territories of the same State if the conveyance has contacts with the territory of any other State on its voyage but only as regards those contacts.

(b) in the case of a traveller, a voyage involving entry into the territory of a State other than the territory of the State in which that traveller commences the voyage.

See also: ‘in transit’.

In transit: a *traveller* undertaking a voyage involving entry into the territory of a State other than the territory of the State in which that traveller commences or ends the voyage. Can be said of a passenger or *traveller* at an airport who is there simply to change flight and is therefore not required to go through customs or immigration formalities. However each country has its own requirements regarding exemption of such formalities and duration of transit. See also: ‘international voyage’.

Invasive: the puncture or incision of the skin or insertion of an instrument or foreign material into the body or the examination of a body cavity.^[[62]](#footnote-62)^ See also: ‘non-invasive’.

Isolation: separation of ill or contaminated persons or affected baggage, containers, conveyances, goods or postal parcels from others in such a manner as to prevent the spread of infection or contamination.^[[63]](#footnote-63)^ See also: ‘detention’.

## **J**

## **K**

## **L**

Legislation: a set of laws enacted by a body with the power to make laws, and includes statutes, regulation and codes recognized as enforceable in accordance with the rules of each country’s legal system.

Local level: limited to a specific geographical area within a country.

## **M**

Mass gathering: A gathering together of a large number of people in one place. Large crowds are commonly associated with leisure events but may occur at religious festivals, parades, and demonstrations and during public disorder. A figure of 1000 has been suggested to constitute a mass gathering.^[[64]](#footnote-64)^

Medical examination: the preliminary assessment of a person by an authorised health worker or by a person under the direct supervision of the competent authority, to determine the person’s health status and potential public health risk to others, and may include the scrutiny of health documents, and a physical examination when justified by the circumstances of the individual case.^[[65]](#footnote-65)^ See also: ‘invasive’, ‘non-invasive’.

Medical treatment: Pharmaceutical and non-pharmaceutical measures that are prescribed or given by physicians and registered professional para-medical personnel to an individual affected by a certain condition.

Member-States (of EU): (of EU): [Austria](http://europa.eu/abc/european_countries/eu_members/austria/index_en.htm), [Belgium](http://europa.eu/abc/european_countries/eu_members/belgium/index_en.htm), [Bulgaria](http://europa.eu/abc/european_countries/eu_members/bulgaria/index_en.htm), [Cyprus](http://europa.eu/abc/european_countries/eu_members/cyprus/index_en.htm), [Czech Republic](http://europa.eu/abc/european_countries/eu_members/czechrepublic/index_en.htm), [Denmark](http://europa.eu/abc/european_countries/eu_members/denmark/index_en.htm), [Estonia](http://europa.eu/abc/european_countries/eu_members/estonia/index_en.htm), [Finland](http://europa.eu/abc/european_countries/eu_members/finland/index_en.htm), [France](http://europa.eu/abc/european_countries/eu_members/france/index_en.htm), [Germany](http://europa.eu/abc/european_countries/eu_members/germany/index_en.htm), [Greece](http://europa.eu/abc/european_countries/eu_members/greece/index_en.htm), [Hungary](http://europa.eu/abc/european_countries/eu_members/hungary/index_en.htm), [Ireland](http://europa.eu/abc/european_countries/eu_members/ireland/index_en.htm), [Italy](http://europa.eu/abc/european_countries/eu_members/italy/index_en.htm), [Latvia](http://europa.eu/abc/european_countries/eu_members/latvia/index_en.htm), [Lithuania](http://europa.eu/abc/european_countries/eu_members/lithuania/index_en.htm), [Luxembourg](http://europa.eu/abc/european_countries/eu_members/luxembourg/index_en.htm), [Malta](http://europa.eu/abc/european_countries/eu_members/malta/index_en.htm), [Netherlands](http://europa.eu/abc/european_countries/eu_members/netherlands/index_en.htm), [Poland](http://europa.eu/abc/european_countries/eu_members/poland/index_en.htm), [Portugal](http://europa.eu/abc/european_countries/eu_members/portugal/index_en.htm),, [Romania](http://europa.eu/abc/european_countries/eu_members/romania/index_en.htm), [Slovakia](http://europa.eu/abc/european_countries/eu_members/slovakia/index_en.htm), [Slovenia](http://europa.eu/abc/european_countries/eu_members/slovenia/index_en.htm), [Spain](http://europa.eu/abc/european_countries/eu_members/spain/index_en.htm), [Sweden](http://europa.eu/abc/european_countries/eu_members/sweden/index_en.htm), [United Kingdom](http://europa.eu/abc/european_countries/eu_members/unitedkingdom/index_en.htm).

Candidate countries: [Croatia](http://europa.eu/abc/european_countries/candidate_countries/croatia/index_en.htm), [Former Yugoslav Republic of Macedonia](http://europa.eu/abc/european_countries/candidate_countries/fyrom/index_en.htm), [Turkey](http://europa.eu/abc/european_countries/candidate_countries/turkey/index_en.htm).

Monitoring (of health): the continuous investigation of a given population or subpopulation, and its environment, to detect changes in the prevalence of a disease or characteristics of a pathogenic agent. ^[[66]](#footnote-66)^

## **N**

National contingency plan: see ‘National Preparedness Plan’.

National IHR Focal Point: the national centre, designated by each State Party, which shall be accessible at all times for communication with WHO IHR Contact Points under IHR, 2005.^[[67]](#footnote-67)^

National level: covering the geographical territory of a State or a country.

National plan: a national influenza preparedness plan.

Non-essential workers: In the context of the PHLawFlu scanario, this refers to those workers who are not essential for the continuity of business activities in any given organisation. See also: ‘project’.

Non-invasive: A [medical procedure](http://en.wikipedia.org/wiki/Medical_procedure) which does not penetrate [mechanically](http://en.wikipedia.org/wiki/Mechanics), nor break the [skin](http://en.wikipedia.org/wiki/Skin) or a [body cavity](http://en.wikipedia.org/wiki/Body_cavity), i.e., it doesn't require an ([invasive](http://en.wikipedia.org/wiki/Invasive_%28medical%29)) [incision](http://en.wikipedia.org/wiki/Incision) into the body or the removal of [biological tissue](http://en.wikipedia.org/wiki/Biological_tissue).^[[68]](#footnote-68)^ Defined in the IHR 2005 as: medical examination of the ear, nose and mouth, temperature assessment using an ear, oral or cutaneous thermometer, or thermal imaging; medical inspection; auscultation; external palpation; retinoscopy; external collection of urine, faeces or saliva samples; external measurement of blood pressure; electrocardiography.^[[69]](#footnote-69)^ See also: ‘invasive’.

Non-national: a person who is not a citizen of the country in which he is physically placed.

Non-EU countries: countries not members of the European Union.

Nordic agreement: Treaty of Cooperation between Denmark, Finland, Iceland, Norway and Sweden (the Helsinki Treaty) signed on 23 March 1962 and entered into force on 1 July 1962.  The Nordic Council was established in 1952, and Council rules are set out in the Helsinki Agreement1962. By the treaty the parties undertake "to seek to preserve and further develop co-operation between [their] nations in the legal, cultural, social and financial areas as well as in matters relating to transport and protection of the environment". More recently, it has included a binding commitment on foreign and security policy issues. The original text has been amended by Agreements signed on 13 February 1971, 11 March 1974, 15 June 1983, 6 May 1985, 21 August 1991, 18 March 1993, and 29 September 1995. ^[[70]](#footnote-70)^

## **O**

Offence: the breaking of an enforceable rule or law.

## **P**

Pandemic: The worldwide outbreak of a disease in humans in numbers clearly in excess of normal.^[[71]](#footnote-71)^ See also: ‘WHO phases’.

Pandemic influenza: occurs when a new strain of influenza virus emerges, spreading around the world and infecting many people at once. An influenza virus capable of causing a pandemic is one against which people have no natural immunity, can easily spread from person to person, and is capable of causing severe disease.^[[72]](#footnote-72)^ See also: ‘WHO phases’.

### Personal data:

- any information relating to an identified or identifiable natural person.^[[73]](#footnote-73)^

- any information relating to an identified or identifiable natural person (‘data subject’); an identifiable person is one who can be identified, directly or indirectly, in particular by reference to an identification number or to one or more factors specific to his physical, physiological, mental, economic, cultural or social identity.^[[74]](#footnote-74)^

See also: ‘processing of personal data’.

Personal data Controller: the natural or legal person, public authority, agency or any other body which alone or jointly with others determines the purposes and means of the processing of personal data; where the purposes and means of processing are determined by national or community laws or regulations, the controller or the specific criteria for his nomination may be designated by national or community law.^[[75]](#footnote-75)^

Personal data filing system: any structured set of personal data which are accessible according to specific criteria, whether centralized, decentralized or dispersed on a functional or geographical basis.^[[76]](#footnote-76)^

Personal identification information: any piece of information likely to identify formally a designated person, such as name, date and place of birth, address, etc.

Person enjoying the Community right of free movement^[[77]](#footnote-77)^**:**

(a) *European Union citizens* within the meaning of Article 17(OJ L 158, 30.4.2004, p. 77) of the Treaty, and third-country nationals who are members of the family of a Union citizen exercising his or her right to free movement to whom Directive 2004/58/EC of the European Parliament and of the Council of 29 April 2004 on the right of citizens of the Union and their family members to move and reside freely within the territory of the Member States (OJ L 158, 30.4.2004, p. 77) applies;

(b) *third-country nationals and their family members*, whatever their nationality, who, under agreements between the Community and its Member States, on the one hand, and those third countries, on the other hand, enjoy rights of free movement equivalent to those of Union citizens.

Personal protective equipment: all equipment which is intended to be worn or held by a person at work and which protects him against one or more risks to his health or safety, e.g. gloves, eye protection, and face mask. ^[[78]](#footnote-78)^

Phase: see: “WHO Phases”

Point of entry: a passage for international entry or exit of travellers, baggage, cargo, containers, conveyances, goods and postal parcels as well as agencies and areas providing services to them on entry or exit.^[[79]](#footnote-79)^

Population screening: see “screening”.

Power: legal authorization to decide or to implement measures, including measures to control communicable diseases or limit their spread.

Prevention and control of communicable diseases: the range of measures, including epidemiological investigations, undertaken by the competent public health authorities to prevent and stop the spread of communicable diseases.^[[80]](#footnote-80)^

Processing of personal data: any operation or set of operations which is performed upon personal data, whether or not by automatic means, such as collection, recording, organization, storage, adaptation or alteration, retrieval, consultation, use, disclosure by transmission, dissemination or otherwise making available, alignment or combination, blocking, erasure or destruction.^[[81]](#footnote-81)^ See also: ‘personal data’.

Processor: a natural or legal person, public authority, agency or any other body which processes personal data on behalf of the controller.^[[82]](#footnote-82)^ See also: ‘personal data’; ‘personal data controller’.

Project (the): the PHLaw Flu Project ([www.ephln.org](http://www.ephln.org)). The scenario and workshops are part of the work of the PHLawFlu prohect.

Prophylactic measures: medical measures to defend against or prevent disease. ^[[83]](#footnote-83)^

Prophylaxis: medical measures designed to preserve health (of an individual or of society) and prevent the spread of disease.^[[84]](#footnote-84)^

Public health emergency of international concern: an extraordinary event which is determined, as provided in the IHR, 2005*^[[85]](#footnote-85)^*:

1. to constitute a public health risk to other States through the international spread of disease and
2. to potentially require a coordinated international response.

Public health observation: the monitoring of the health status of a traveller over time for the purpose of determining the risk of disease transmission.^[[86]](#footnote-86)^

Public health risk: the likelihood of an event that may affect adversely the health of human populations, with an emphasis on one which may spread internationally or may present a serious and direct danger.^[[87]](#footnote-87)^

Public places: any street, alley, park, public building, any place of business or assembly open to or frequented by the public, and any other place which is open to the public view, or to which the public has access.^[[88]](#footnote-88)^

## **Q**

Quarantine: the restriction of activities and/or separation from others of suspect persons who are not ill or of suspect baggage, containers, conveyances or goods in such a manner as to prevent the possible spread of infection or contamination.^[[89]](#footnote-89)^

## **R**

Recipient (of personal data): a natural or legal person, public authority, agency or any other body to whom data are disclosed, whether a third party or not; however, authorities which may receive data in the framework of a particular inquiry shall not be regarded as recipients.^[[90]](#footnote-90)^

Refugee: someone who has a well-founded fear of being persecuted only for one or more of five defined reasons: race, religion, nationality, membership of a particular social group and political opinion. The fear must be such that it makes the applicant unwilling or unable to avail him or herself of the protection of the country of nationality.^[[91]](#footnote-91)^

Regional level: for the purposes of the PHLawFlu project, a region is a geographical area with a recognised level of political autonomy and law-making power, that forms part of a wider state or country. See also: ‘project’.

Regular ferry connection: any ferry connection between the same two or more ports situated in the territory of the Schengen Member States, not calling at any ports outside the territory of the Member States and consisting of the transport of passengers and vehicles according to a published timetable.^[[92]](#footnote-92)^

### Regulations:

1. IHR, 2005.^[[93]](#footnote-93)^  See: ‘IHR’.
2. See also the separate ‘Meaning of law’ document

Repatriation: The act of a person in returning from a country where he is a non-citizen to his/her country of origin.

Required: The aim of our study is to identify compulsory powers and measures. Therefore, we use the terminology ‘required’ or ‘require’ which is stronger than ‘permit’ and has more relevance in this context.

Requisition: the power of a national, regional or local government, or agency of government, in a situation of apprehended emergency, to require private property, goods and persons to be used for public purposes in relation to that emergency.

Resident: a person entitled to right of residence or granted a *residence permit* to stay legally in a country.

For the Schengen area, *‘residence permit’* means^[[94]](#footnote-94)^:

(a) all residence permits issued by the Member States according to the uniform format laid down by Council Regulation (EC) No 1030/2002 of 13 June 2002 laying down a uniform format for residence permits for third country nationals (2);

(b) all other documents issued by a Member State to third country nationals authorising a stay in, or re-entry into, its territory, with the exception of temporary permits issued pending examination of a first application for a residence permit as referred to in point (a) or an application for asylum.

Residential establishment: an establishment other than a private home which provides accommodation, and possibly also essential services, for a defined category of persons (older people, children etc.).

Restriction: for the purposes of the PHLawFlu project:

*Of movement:* limitation of an affected person’s movements to/from a place, in order to control or limit spread of disease.

*Of contact:* limitation of a person’s contact with other persons in order to control or limit spread of disease.

See also: ‘project’.

Review: re-examination of a case, especially by a superior court.

Right of appeal: the opportunity of a hearing before a superior judge/court to challenge the decision of an inferior court or to challenge the decision of a person with legal authority to make an enforceable order. In the context of the PHLawFlu project the right of appeal will be relevant to an order for detention or other compulsory measures such as treatment, vaccination, prophylaxis, requisition etc.

## **S**

Sanction: a penalty or punishment.

Sanitary cordon: see “cordon sanitaire”.

Schengen agreement: Regulation (EC) No 562/2006 of the European Parliament and of the Council of 15 March 2006 establishing a Community Code on the rules governing the movement of persons across borders (Schengen Borders Code).^[[95]](#footnote-95)^

Screening: Screening is a public health service in which individuals or members of a defined population are tested, to identify those individuals who are more likely to be helped than harmed by further tests or treatment to reduce the risk of a disease or its complications.^[[96]](#footnote-96)^

*Entry screening:* screening carried at borders and points of entry of persons coming from another country and wishing to enter the country undertaking the screening.

*Exit screening:* screening at borders and points of exit (such as international airports) of persons travelling out from the country undertaking the screening.

See also: ‘invasive’ and ‘non-invasive’.

Seasonal: Relating to or characteristic of a particular season of the year

Seasonal influenza: a respiratory infection caused by human influenza viruses which circulate and affect populations on a seasonal basis. Most people have some immunity, and a vaccine is available. This is also known as the common flu or winter flu.^[[97]](#footnote-97)^ See also: ‘avian influenza’, ‘pandemic influenza’, and ‘influenza’.

Serious threat to public policy or internal security: (in relation to EU states): Regulation (EC) No 562/2006 of 15 March 2006: ‘Where there is a serious threat to public policy or internal security, a Member State may exceptionally reintroduce border control at its internal borders for a limited period of no more than 30 days or for the foreseeable duration of the serious threat if its duration exceeds the period of 30 days, in accordance with the procedure laid down in Article 24 or, in urgent cases, with that laid down in Article 25. The scope and duration of the temporary reintroduction of border control at internal borders shall not exceed what is strictly necessary to respond to the serious threat’.^[[98]](#footnote-98)^

Source: the place, thing, person, circumstance, etc that contamination/contact with a virus begins or develops from; the origin.

Specimen: see ‘biological sample’.

Stop list: list of names and contact details of all passengers on a plane (or any other means of travel) which must be communicated by crew to authorities upon request.

Surveillance: the systematic ongoing collection, collation and analysis of data for public health purposes and the timely dissemination of public health information for assessment and public health response as necessary.^[[99]](#footnote-99)^

Suspect: those persons, baggage, cargo, containers, conveyances, goods or postal parcels considered by a State Party as having been exposed, or possibly exposed, to a public health risk and that could be a possible source of spread of disease.^[[100]](#footnote-100)^

## **T**

Third-country national: any person who is not a Union citizen within the meaning of Article 17(OJ L 158, 30.4.2004, p. 77) of the Treaty and who is not ‘persons enjoying the Community right of free movement’.^[[101]](#footnote-101)^

Third-party: (in the context of personal data)**:** any natural or legal person, public authority, agency or any controller, the processor and the persons who, under the direct authority of the controller or the processor, are authorized to process personal data.^[[102]](#footnote-102)^

Threat to public health: any disease with epidemic potential as defined by the International Health Regulations of the World Health Organization and other infectious diseases or contagious parasitic diseases if they are the subject of protection provisions applying to nationals of the Member States.^[[103]](#footnote-103)^

Trace: see: ‘contact tracing’.

Transit: see: ‘in transit’.

Transmission: any means of spreading infectious disease to or among people. ^[[104]](#footnote-104)^

Traveller: a natural person undertaking an international voyage.^[[105]](#footnote-105)^

## **U**

Undocumented immigrant: a person who has entered a country illegally or who is illegally within a country.

Unlicensed product: (prophylaxis, medicine or vaccine) is one that does not have a product license.^[[106]](#footnote-106)^

Unlicensed staff: a person having no official licence to practise a profession. Licence/licensure is a permission granted to an individual or organisation by a competent authority, usually public (e.g., a state government), to engage lawfully in a practice, occupation, or activity. Licensure is the process by which the licence is granted. It is usually granted on the basis of examination and/or proof of education rather than on measures of performance. A licence is usually permanent but may be conditional on annual payment of a fee, proof of continuing education, or proof of competence.^[[107]](#footnote-107)^ Said of persons (such as medical students, etc.) not entitled officially to practise medicine or to provide health care, but who are required to provide health care under specific circumstances such as a pandemic.

Union citizen: any natural person having the nationality of a Member State of the European Union.^[[108]](#footnote-108)^

## **V**

Vaccination (inoculation): a specific prophylactic measure for the purpose of inducing or maintaining the insusceptibility of an organism to an infectious disease by injecting a vaccine.^[[109]](#footnote-109)^ The successful immunisation of susceptible animals or persons through the administration of a vaccine comprising antigens appropriate to the disease to be controlled.^[[110]](#footnote-110)^

Vaccine: A substance produced from an infectious organism that stimulates an immune response (but not disease) thereby protecting against subsequent infection by that organism.^[[111]](#footnote-111)^

Vaccine coverage: The proportion or percentage of persons that have received a vaccine

among all individuals in a particular group who are eligible to receive the vaccine. ^[[112]](#footnote-112)^

Verification: the provision of information by a State Party to WHO confirming the status of an event within the territory or territories of that State Party.^[[113]](#footnote-113)^

Visa: a permit or authorisation stamped into a passport or similar document, allowing the holder to enter or leave the issuing country.

Visitor: someone who visits a country.

Vital services: In the context of the PHLawFlu project, this refers to those services which are essential for the continuity of basic societal activities/functions. Vital service enterprises include those providing water, food, medicines, energy and transport. See also: ‘project’.

Vital workers: In the context of the PHLawFlu project, this refers to those workers who are essential to the operation of vital service enterprises such as water, energy, etc. See also: ‘essential workers’; ‘vital services’; ‘project’.

Voluntary: an act performed by free choice and not under obligation or compulsion, for example self-quarantining other than in response to a quarantine order.

## **W**

WHO: the World Health Organization.

WHO phases: WHO uses a series of six phases of pandemic alert as a system for informing the world of the seriousness of the threat and of the need to launch progressively more intense preparedness activities. The designation of phases, including decisions on when to move from one phase to another, is made by the Director-General of WHO. Each phase of alert coincides with a series of recommended activities to be undertaken by WHO, the international community, governments, and industry. Changes from one phase to another are triggered by factors such as the epidemiological behaviour of the disease and the characteristics of circulating viruses.^[[114]](#footnote-114)^

| **Pandemic periods and WHO global pandemic phases** | | **National stages** |
| --- | --- | --- |
| *Inter-pandemic period* | **Phase 1**.  No new influenza virus subtypes have been detected in humans. An influenza virus subtype that has caused human infection may be present in animals. If present in animals, the risk of human infection or disease is considered to be low. |  |
|  |  |  |
|  | **Phase 2**.  No new influenza virus subtypes have been detected in humans. However, links with affected country. a circulating animal influenza virus subtype poses a substantial risk of human disease. | Not affected |
|  |  | Affected or extensive travel/trade links with affected countries |
| *Pandemic alert period* | **Phase 3**  Human infection(s) with a new subtype, but no human to human spread , or at most rare instances of spread to a close contact | Not affected |
|  |  | Affected or extensive travel/trade links with affected countries |
|  | **Phase 4**  Small cluster(s) with limited human to human transmission but spread is highly localized, suggesting that the virus is not well adapted to humans | Not affected |
|  |  | Affected or extensive travel/trade links with affected countries |
|  | **Phase 5**  Larger cluster(s) but human to human spread still localized, suggesting that the virus is becoming increasingly better adapted to humans, but may not yet be fully transmissible (substantial pandemic risk) | Not affected |
|  |  | Affected or extensive travel/trade links with affected countries |
| *Pandemic period* | **Phase 6**  Pandemic phase: increased and sustained transmission in general population | Not yet affected |
|  |  | Affected or extensive travel/trade links with affected countries |
|  |  | Subsided |
|  |  | Next wave |
| *Post-pandemic period* | Return to inter-pandemic period |  |

**NB**: W.H.O. is in the process of revising its pandemic preparedness guidance. Therefore these phases are likely to change in the near future.

WHO IHR Contact Point: the unit within WHO which shall be accessible at all times for communications with the National IHR Focal Point.^[[115]](#footnote-115)^

Within a country: visitors, residents, undocumented immigrants and refugees who have passed though border control.

Workshop: For the purposes of the PHLawFlu project, a meeting of invited state representatives/rapporteurs to examine legal issues arising from the project disease scenario. See also: ‘project’.

## **X**

## **Y**

## **Z**

1. http://www.pandemicflu.gov/faq/avianinfluenza/ [↑](#footnote-ref-1)
2. http://www.hpa.org.uk/infections/topics_az/influenza/pandemic/pandemicflufaq_virus.htm [↑](#footnote-ref-2)
3. http://www.who.int/mediacentre/factsheets/fs211/en/index.html [↑](#footnote-ref-3)
4. http://www.who.int/csr/disease/avian_influenza/phase/en/index.html [↑](#footnote-ref-4)
5. http://www.cdc.gov/flu/about/viruses/index.htm [↑](#footnote-ref-5)
6. http://www.pandemicflu.gov/faq/pandemicinfluenza/ [↑](#footnote-ref-6)
7. Such as: International health Regulations (IHR 2005) which are binding on all the countries that are the subject of the PHLawFlu project; Decision N° 2119/98/EC of 24 September 1998 setting up a network for the epidemiological surveillance and control of communicable diseases in the Community; Regulation (EC) N° 851/2004 of 21 April 2004 establishing a European centre for disease prevention and control; Regulation (EC) No 562/2006 on Schengen Borders Code; Directive 2004/82/EC of 29 April 2004 on the obligation of carriers to communicate passenger data. (**NB**: UK and Ireland are parties to this Directive); Decision 2003/534/EC of 17 July 2003 amending Decision No 2119/98/EC of the European Parliament and of the Council and Decision 2000/96/EC as regards communicable diseases listed in those decisions and amending Decision 2002/253/EC as regards the case definitions for communicable diseases; Directive 95/46/EC of 24 October 1995 on the protection of individuals with regard to the processing of personal data and on the free movement of such data; and many others. [↑](#footnote-ref-7)
8. World Health Organization, *International health regulations*, 2005, (<http://www.who.int/csr/ihr/en/index.html>, retrieved 13 December 2007). [↑](#footnote-ref-8)
9. World Health Organization, *International health regulations*, 2005, (<http://www.who.int/csr/ihr/en/index.html>, retrieved 13 December 2007). [↑](#footnote-ref-9)
10. <http://www.pandemicflu.gov/glossary/index.html>, (retrieved 1st May, 2008). [↑](#footnote-ref-10)
11. http://stats.oecd.org/glossary/detail.asp?ID=7222 [↑](#footnote-ref-11)
12. Regulation (EC) No 562/2006 of the European Parliament and of the Council of 15 March 2006 establishing a Community Code on the rules governing the movement of persons across borders (Schengen Borders Code)

    <http://eur-lex.europa.eu/LexUriServ/LexUriServ.do?uri=OJ:L:2006:105:0001:0032:EN:PDF>, retrieved 28 January 2008). [↑](#footnote-ref-12)
13. Regulation (EC) No 562/2006 of the European Parliament and of the Council of 15 March 2006 establishing a Community Code on the rules governing the movement of persons across borders (Schengen Borders Code)

    <http://eur-lex.europa.eu/LexUriServ/LexUriServ.do?uri=OJ:L:2006:105:0001:0032:EN:PDF>, retrieved 28 January 2008). [↑](#footnote-ref-13)
14. Directive 2004/82/EC of 29 April 2004 on the obligation of carriers to communicate passenger data, article 2, (<http://eur-lex.europa.eu/LexUriServ/LexUriServ.do?uri=OJ:L:2004:261:0024:0027:EN:PDF>, retrieved 6 February 2008). [↑](#footnote-ref-14)
15. Regulation (EC) No 562/2006 of the European Parliament and of the Council of 15 March 2006 establishing a Community Code on the rules governing the movement of persons across borders (Schengen Borders Code)

    <http://eur-lex.europa.eu/LexUriServ/LexUriServ.do?uri=OJ:L:2006:105:0001:0032:EN:PDF>, retrieved 28 January 2008). [↑](#footnote-ref-15)
16. Regulation (EC) No 562/2006 of the European Parliament and of the Council of 15 March 2006 establishing a Community Code on the rules governing the movement of persons across borders (Schengen Borders Code)

    <http://eur-lex.europa.eu/LexUriServ/LexUriServ.do?uri=OJ:L:2006:105:0001:0032:EN:PDF>, retrieved 28 January 2008). [↑](#footnote-ref-16)
17. World Health Organization, *Ethical considerations in developing a public health response to pandemic influenza*, Geneva, World Health Organization, 2007,

    (<http://www.who.int/csr/resources/publications/WHO_CDS_EPR_GIP_2007_2/en/index.html>, retrieved 4 February 2008). [↑](#footnote-ref-17)
18. Regulation (EC) No 562/2006 of the European Parliament and of the Council of 15 March 2006 establishing a Community Code on the rules governing the movement of persons across borders (Schengen Borders Code)

    <http://eur-lex.europa.eu/LexUriServ/LexUriServ.do?uri=OJ:L:2006:105:0001:0032:EN:PDF>, retrieved 28 January 2008). [↑](#footnote-ref-18)
19. Regulation (EC) No 562/2006 of the European Parliament and of the Council of 15 March 2006 establishing a Community Code on the rules governing the movement of persons across borders (Schengen Borders Code)

    <http://eur-lex.europa.eu/LexUriServ/LexUriServ.do?uri=OJ:L:2006:105:0001:0032:EN:PDF>, retrieved 28 January 2008). [↑](#footnote-ref-19)
20. Regulation (EC) No 562/2006 of the European Parliament and of the Council of 15 March 2006 establishing a Community Code on the rules governing the movement of persons across borders (Schengen Borders Code)

    <http://eur-lex.europa.eu/LexUriServ/LexUriServ.do?uri=OJ:L:2006:105:0001:0032:EN:PDF>, retrieved 28 January 2008). [↑](#footnote-ref-20)
21. Directive 2004/82/EC of 29 April 2004 on the obligation of carriers to communicate passenger data, article 2, (<http://eur-lex.europa.eu/LexUriServ/LexUriServ.do?uri=OJ:L:2004:261:0024:0027:EN:PDF>, retrieved 6 February 2008). [↑](#footnote-ref-21)
22. <http://www.fao.org/avianflu/en/glossary.html> [↑](#footnote-ref-22)
23. http://www.fao.org/avianflu/en/glossary.html [↑](#footnote-ref-23)
24. See list in Decision 2003/534/EC of 17 July 2003 amending Decision No 2119/98/EC of the European Parliament and of the Council and Decision 2000/96/EC as regards communicable diseases listed in those decisions and amending Decision 2002/253/EC as regards the case definitions for communicable diseases (<http://eur-lex.europa.eu/smartapi/cgi/sga_doc?smartapi!celexplus!prod!DocNumber&type_doc=Decision&an_doc=2003&nu_doc=534&lg=en> ,retrieved 6 February 2008). [↑](#footnote-ref-24)
25. Directive 95/46/EC of 24 October 1995 on the protection of individuals with regard to the processing of personal data and on the free movement of such data, (<http://ec.europa.eu/justice_home/fsj/privacy/docs/95-46-ce/dir1995-46_part1_en.pdf> , retrieved 6 February 2008). [↑](#footnote-ref-25)
26. Adapted from http://www.cdc.gov/tb/pubs/ssmodules/glos%206-9.htm [↑](#footnote-ref-26)
27. <http://cancerweb.ncl.ac.uk/cgi-bin/omd?contact+tracing>. [↑](#footnote-ref-27)
28. World Health Organization, *International health regulations*, 2005, (<http://www.who.int/csr/ihr/en/index.html>, retrieved 13 December 2007). [↑](#footnote-ref-28)
29. World Health Organization, *International health regulations*, 2005, (<http://www.who.int/csr/ihr/en/index.html>, retrieved 13 December 2007). [↑](#footnote-ref-29)
30. World Health Organization, *International health regulations*, 2005, (<http://www.who.int/csr/ihr/en/index.html>, retrieved 13 December 2007). [↑](#footnote-ref-30)
31. WHO Interim Protocol: Rapid operations to contain the initial emergence of pandemic influenza, October 2007, (<http://www.who.int/csr/disease/avian_influenza/guidelines/RapidContProtOct15.pdf> , retrieved 13 March 2008). [↑](#footnote-ref-31)
32. <http://www.answers.com/topic/court-order?cat=biz-fin>. [↑](#footnote-ref-32)
33. World Health Organization, *International health regulations*, 2005, (<http://www.who.int/csr/ihr/en/index.html>, retrieved 13 December 2007). [↑](#footnote-ref-33)
34. World Health Organization, *International health regulations*, 2005, (<http://www.who.int/csr/ihr/en/index.html>, retrieved 13 December 2007). [↑](#footnote-ref-34)
35. <http://ec.europa.eu/dgs/health_consumer/index_en.htm>. [↑](#footnote-ref-35)
36. World Health Organization, *International health regulations*, 2005, (<http://www.who.int/csr/ihr/en/index.html>, retrieved 13 December 2007). [↑](#footnote-ref-36)
37. http://www.jhsph.edu/publichealthexperts/Glossary.htm [↑](#footnote-ref-37)
38. World Health Organization, *International health regulations*, 2005, (<http://www.who.int/csr/ihr/en/index.html>, retrieved 13 December 2007). [↑](#footnote-ref-38)
39. <http://secretariat.efta.int/Web/EuropeanEconomicArea/introduction> . [↑](#footnote-ref-39)
40. See EFTA website; <http://www.efta.int/> [↑](#footnote-ref-40)
41. http://www.pandemicflu.gov/glossary/index.html#C [↑](#footnote-ref-41)
42. http://www.cdc.gov/tb/pubs/ssmodules/glos%206-9.htm [↑](#footnote-ref-42)
43. World Health Organization, *International health regulations*, 2005, (<http://www.who.int/csr/ihr/en/index.html>, retrieved 13 December 2007). [↑](#footnote-ref-43)
44. Directive 2004/58/EC of 29 April 2004 on the right of citizens of the Union and their family members to move and reside freely within the territory of the Member States, (<http://eur-lex.europa.eu/LexUriServ/LexUriServ.do?uri=OJ:L:2004:229:0035:0048:EN:PDF>, retrieved 28 January 2008). [↑](#footnote-ref-44)
45. See Harrap’s 21^st^ Century Dictionary. [↑](#footnote-ref-45)
46. World Health Organization, *International health regulations*, 2005, (<http://www.who.int/csr/ihr/en/index.html>, retrieved 13 December 2007). [↑](#footnote-ref-46)
47. Influenza A (H5N1): WHO Interim Infection Control Guidelines for Health Care Facilities, March 2004, p. 17,

    <http://www.who.int/csr/disease/avian_influenza/guidelines/Guidelines_for_health_care_facilities.pdf> , retrieved 1st May 2008). [↑](#footnote-ref-47)
48. Influenza A (H5N1): WHO Interim Infection Control Guidelines for Health Care Facilities, March 2004, p. 17,

    <http://www.who.int/csr/disease/avian_influenza/guidelines/Guidelines_for_health_care_facilities.pdf> , retrieved 1st May 2008). [↑](#footnote-ref-48)
49. Health insurance. InvestorWords.com. WebFinance, Inc. http://www.investorwords.com/2289/health_insurance.html (accessed: June 3, 2008). [↑](#footnote-ref-49)
50. World Health Organization, *International health regulations*, 2005, (<http://www.who.int/csr/ihr/en/index.html>, retrieved 13 December 2007). [↑](#footnote-ref-50)
51. Regulation (EC) N° 851/2004 of 21 April 2004 establishing a European centre for disease prevention and control, art. 2-(e), <http://eur-lex.europa.eu/LexUriServ/LexUriServ.do?uri=CELEX:32004R0851:EN:HTML>, retrieved 6 February 2008). [↑](#footnote-ref-51)
52. Directive 2004/58/EC of 29 April 2004 on the right of citizens of the Union and their family members to move and reside freely within the territory of the Member States, (<http://eur-lex.europa.eu/LexUriServ/LexUriServ.do?uri=OJ:L:2004:229:0035:0048:EN:PDF>, retrieved 28 January 2008). [↑](#footnote-ref-52)
53. See <http://www.who.int/mediacentre/factsheets/fs211/en/> . [↑](#footnote-ref-53)
54. World Health Organization, *International health regulations*, 2005, (<http://www.who.int/csr/ihr/en/index.html>, retrieved 13 December 2007). [↑](#footnote-ref-54)
55. World Health Organization, *International health regulations*, 2005, (<http://www.who.int/csr/ihr/en/index.html>, retrieved 13 December 2007). [↑](#footnote-ref-55)
56. World Health Organization, *Western Pacific Region, Exercise Development Guide For Validating Influenza Pandemic Preparedness*, February 2006, (<http://www.wpro.who.int/NR/rdonlyres/DA340E3E-D27E-47A6-9833-452E7AAC9ED5/0/EDTedDRAFT1ExerciseDevelopmentGuide.pdf>, retrieved 19 January 2008). [↑](#footnote-ref-56)
57. Definitions retrieved from <http://www.wipo.int/freepublications/en/intproperty/895/wipo_pub_895.pdf>, June 3 2008. [↑](#footnote-ref-57)
58. Regulation (EC) No 562/2006 of the European Parliament and of the Council of 15 March 2006 establishing a Community Code on the rules governing the movement of persons across borders (Schengen Borders Code)

    <http://eur-lex.europa.eu/LexUriServ/LexUriServ.do?uri=OJ:L:2006:105:0001:0032:EN:PDF>, retrieved 28 January 2008). [↑](#footnote-ref-58)
59. Adapted from World Health Organization, *International health regulations*, 2005, (http://[www.who.int/ihr](http://www.who.int/ihr), retrieved 15 September 2008). [↑](#footnote-ref-59)
60. For more details see *International Health Regulations (2005): Areas for work implementation*, available at: http://www.who.int/csr/ihr/finalversion9Nov07.pdf [↑](#footnote-ref-60)
61. Regulation (EC) No 562/2006 of the European Parliament and of the Council of 15 March 2006 establishing a Community Code on the rules governing the movement of persons across borders (Schengen Borders Code)

    <http://eur-lex.europa.eu/LexUriServ/LexUriServ.do?uri=OJ:L:2006:105:0001:0032:EN:PDF>, retrieved 28 January 2008). [↑](#footnote-ref-61)
62. World Health Organization, *International health regulations*, 2005, (<http://www.who.int/csr/ihr/en/index.html>, retrieved 13 December 2007). [↑](#footnote-ref-62)
63. World Health Organization, *International health regulations*, 2005, (<http://www.who.int/csr/ihr/en/index.html>, retrieved 13 December 2007). [↑](#footnote-ref-63)
64. Hodgetts T J*,* Cooke M W, The largest mass gathering. *BMJ* 1999;318;957-958, <http://bmj.bmjjournals.com/cgi/content/full/318/7189/957>, retrieved on 3 June 2008, referring to Sanders A, Criss E, Steckl P, Meislin H, Raife J, Allen D. An analysis of medical care at mass gatherings. *Ann Emerg Med* 1986; 15: 515-519[[Medline]](http://bmj.bmjjournals.com/cgi/external_ref?access_num=3963529&link_type=MED). [↑](#footnote-ref-64)
65. World Health Organization, *International health regulations*, 2005, (<http://www.who.int/csr/ihr/en/index.html>, retrieved 13 December 2007). [↑](#footnote-ref-65)
66. http://www.fao.org/avianflu/en/glossary.html [↑](#footnote-ref-66)
67. World Health Organization, *International health regulations*, 2005, (<http://www.who.int/csr/ihr/en/index.html>, retrieved 13 December 2007). [↑](#footnote-ref-67)
68. http://en.wikipedia.org/wiki/Noninvasive [↑](#footnote-ref-68)
69. World Health Organization, *International health regulations*, 2005, (<http://www.who.int/csr/ihr/en/index.html>, retrieved 13 December 2007). [↑](#footnote-ref-69)
70. For more information, see the website:<http://www.norden.org/avtal/helsingfors/uk/3-2-2-hfors.asp?lang>=. [↑](#footnote-ref-70)
71. <http://www.pandemicflu.gov/glossary/index.html> ,(retrieved 1st May, 2008). [↑](#footnote-ref-71)
72. http://www.fao.org/avianflu/en/glossary.html [↑](#footnote-ref-72)
73. World Health Organization, *International health regulations*, 2005, (<http://www.who.int/csr/ihr/en/index.html>, retrieved 13 December 2007). [↑](#footnote-ref-73)
74. Directive 95/46/EC of 24 October 1995 on the protection of individuals with regard to the processing of personal data and on the free movement of such data, (<http://ec.europa.eu/justice_home/fsj/privacy/docs/95-46-ce/dir1995-46_part1_en.pdf> , retrieved 6 February 2008). [↑](#footnote-ref-74)
75. Directive 95/46/EC of 24 October 1995 on the protection of individuals with regard to the processing of personal data and on the free movement of such data, (<http://ec.europa.eu/justice_home/fsj/privacy/docs/95-46-ce/dir1995-46_part1_en.pdf> , retrieved 6 February 2008). [↑](#footnote-ref-75)
76. Directive 95/46/EC of 24 October 1995 on the protection of individuals with regard to the processing of personal data and on the free movement of such data, (<http://ec.europa.eu/justice_home/fsj/privacy/docs/95-46-ce/dir1995-46_part1_en.pdf> , retrieved 6 February 2008). [↑](#footnote-ref-76)
77. Regulation (EC) No 562/2006 of the European Parliament and of the Council of 15 March 2006 establishing a Community Code on the rules governing the movement of persons across borders (Schengen Borders Code)

    <http://eur-lex.europa.eu/LexUriServ/LexUriServ.do?uri=OJ:L:2006:105:0001:0032:EN:PDF>, retrieved 28 January 2008). [↑](#footnote-ref-77)
78. http://www.hse.gov.uk/pubns/indg174.pdf [↑](#footnote-ref-78)
79. World Health Organization, *International health regulations*, 2005, (<http://www.who.int/csr/ihr/en/index.html>, retrieved 13 December 2007). [↑](#footnote-ref-79)
80. Decision N° 2119/98/EC of 24 September 1998 setting up a network for the epidemiological surveillance and control of communicable diseases in the Community, article 2, (<http://eur-lex.europa.eu/LexUriServ/LexUriServ.do?uri=CELEX:31998D2119:EN:HTML>, retrieved 6 February 2008). [↑](#footnote-ref-80)
81. Directive 95/46/EC of 24 October 1995 on the protection of individuals with regard to the processing of personal data and on the free movement of such data, (<http://ec.europa.eu/justice_home/fsj/privacy/docs/95-46-ce/dir1995-46_part1_en.pdf> , retrieved 6 February 2008). [↑](#footnote-ref-81)
82. Directive 95/46/EC of 24 October 1995 on the protection of individuals with regard to the processing of personal data and on the free movement of such data, (<http://ec.europa.eu/justice_home/fsj/privacy/docs/95-46-ce/dir1995-46_part1_en.pdf> , retrieved 6 February 2008). [↑](#footnote-ref-82)
83. World Health Organization, *Ethical considerations in developing a public health response to pandemic influenza*, Geneva, World Health Organization, 2007,

    (<http://www.who.int/csr/resources/publications/WHO_CDS_EPR_GIP_2007_2/en/index.html>, retrieved 4 February 2008). [↑](#footnote-ref-83)
84. <http://www.merriam-webster.com/dictionary/prophylaxis> [↑](#footnote-ref-84)
85. World Health Organization, *Western Pacific Region, Exercise Development Guide For Validating Influenza Pandemic Preparedness*, February 2006, (<http://www.wpro.who.int/NR/rdonlyres/DA340E3E-D27E-47A6-9833-452E7AAC9ED5/0/EDTedDRAFT1ExerciseDevelopmentGuide.pdf>, retrieved 19 January 2008). [↑](#footnote-ref-85)
86. World Health Organization, *International health regulations*, 2005, (<http://www.who.int/csr/ihr/en/index.html>, retrieved 13 December 2007). [↑](#footnote-ref-86)
87. World Health Organization, *International health regulations*, 2005, (<http://www.who.int/csr/ihr/en/index.html>, retrieved 13 December 2007). [↑](#footnote-ref-87)
88. <http://www.hillsdalecounty.info/downloads/51disorderly.pdf>. [↑](#footnote-ref-88)
89. World Health Organization, *International health regulations*, 2005, (<http://www.who.int/csr/ihr/en/index.html>, retrieved 13 December 2007). [↑](#footnote-ref-89)
90. Directive 95/46/EC of 24 October 1995 on the protection of individuals with regard to the processing of personal data and on the free movement of such data, (<http://ec.europa.eu/justice_home/fsj/privacy/docs/95-46-ce/dir1995-46_part1_en.pdf> , retrieved 6 February 2008). [↑](#footnote-ref-90)
91. **Council Directive 2004/83/EC of 29 April 2004 on minimum standards for the qualification and status of third country nationals or stateless persons as refugees or as persons who otherwise need international protection and the content of the protection granted, article 1-c;** Official Journal L 304 , 30/09/2004 P. 0012 – 0023,

    <http://eur-lex.europa.eu/LexUriServ/LexUriServ.do?uri=CELEX:32004L0083:EN:HTML> **, retrieved 12 march 2008.** [↑](#footnote-ref-91)
92. Regulation (EC) No 562/2006 of the European Parliament and of the Council of 15 March 2006 establishing a Community Code on the rules governing the movement of persons across borders (Schengen Borders Code)

    <http://eur-lex.europa.eu/LexUriServ/LexUriServ.do?uri=OJ:L:2006:105:0001:0032:EN:PDF>, retrieved 28 January 2008). [↑](#footnote-ref-92)
93. World Health Organization, *International health regulations*, 2005, (<http://www.who.int/csr/ihr/en/index.html>, retrieved 13 December 2007). [↑](#footnote-ref-93)
94. Regulation (EC) No 562/2006 of the European Parliament and of the Council of 15 March 2006 establishing a Community Code on the rules governing the movement of persons across borders (Schengen Borders Code)

    <http://eur-lex.europa.eu/LexUriServ/LexUriServ.do?uri=OJ:L:2006:105:0001:0032:EN:PDF>, retrieved 28 January 2008). [↑](#footnote-ref-94)
95. <http://eur-lex.europa.eu/LexUriServ/LexUriServ.do?uri=OJ:L:2006:105:0001:0032:EN:PDF>, retrieved 28 January 2008). [↑](#footnote-ref-95)
96. http://www.nsc.nhs.uk/whatscreening/whatscreen_ind.htm [↑](#footnote-ref-96)
97. <http://www.pandemicflu.gov/glossary/index.html> ,(retrieved 1st May, 2008). [↑](#footnote-ref-97)
98. Regulation (EC) No 562/2006 of the European Parliament and of the Council of 15 March 2006 establishing a Community Code on the rules governing the movement of persons across borders (Schengen Borders Code), article 23-1, <http://eur-lex.europa.eu/LexUriServ/LexUriServ.do?uri=OJ:L:2006:105:0001:0032:EN:PDF>, retrieved 28 January 2008). [↑](#footnote-ref-98)
99. World Health Organization, *International health regulations*, 2005, (<http://www.who.int/csr/ihr/en/index.html>, retrieved 13 December 2007). [↑](#footnote-ref-99)
100. World Health Organization, *International health regulations*, 2005, (<http://www.who.int/csr/ihr/en/index.html>, retrieved 13 December 2007). [↑](#footnote-ref-100)
101. Regulation (EC) No 562/2006 of the European Parliament and of the Council of 15 March 2006 establishing a Community Code on the rules governing the movement of persons across borders (Schengen Borders Code)

     <http://eur-lex.europa.eu/LexUriServ/LexUriServ.do?uri=OJ:L:2006:105:0001:0032:EN:PDF>, retrieved 28 January 2008). [↑](#footnote-ref-101)
102. Directive 95/46/EC of 24 October 1995 on the protection of individuals with regard to the processing of personal data and on the free movement of such data, (<http://ec.europa.eu/justice_home/fsj/privacy/docs/95-46-ce/dir1995-46_part1_en.pdf> , retrieved 6 February 2008). [↑](#footnote-ref-102)
103. Regulation (EC) No 562/2006 of the European Parliament and of the Council of 15 March 2006 establishing a Community Code on the rules governing the movement of persons across borders (Schengen Borders Code)

     <http://eur-lex.europa.eu/LexUriServ/LexUriServ.do?uri=OJ:L:2006:105:0001:0032:EN:PDF>, retrieved 28 January 2008). [↑](#footnote-ref-103)
104. http://www.jhsph.edu/publichealthexperts/Glossary.htm [↑](#footnote-ref-104)
105. World Health Organization, *International health regulations*, 2005, (<http://www.who.int/csr/ihr/en/index.html>, retrieved 13 December 2007). [↑](#footnote-ref-105)
106. http://www.nhsdirect.nhs.uk/articles/article.aspx?ArticleId=1004 [↑](#footnote-ref-106)
107. Source: Health Care in America: Trends in Utilization. See: <http://www.cdc.gov/nchs/datawh/nchsdefs/licensure.htm> [↑](#footnote-ref-107)
108. Directive 2004/58/EC of 29 April 2004 on the right of citizens of the Union and their family members to move and reside freely within the territory of the Member States, (<http://eur-lex.europa.eu/LexUriServ/LexUriServ.do?uri=OJ:L:2004:229:0035:0048:EN:PDF>, retrieved 28 January 2008). [↑](#footnote-ref-108)
109. Latvia, Epidemiological Safety Law, (with amendments of 30 March 2000), <http://www.ttc.lv/index.php?skip=15&itid=likumi&id=10&tid=50&l=EN> , (retrieved 1^st^ May 2008). [↑](#footnote-ref-109)
110. http://www.fao.org/avianflu/en/glossary.html [↑](#footnote-ref-110)
111. http://www.fao.org/avianflu/en/glossary.html [↑](#footnote-ref-111)
112. http://www.cdc.gov/vaccines/stats-surv/downloads/definitions.pdf [↑](#footnote-ref-112)
113. World Health Organization, *International health regulations*, 2005, (<http://www.who.int/csr/ihr/en/index.html>, retrieved 13 December 2007). [↑](#footnote-ref-113)
114. http://www.who.int/csr/disease/avian_influenza/phase/en/index.html [↑](#footnote-ref-114)
115. World Health Organization, *International health regulations*, 2005, (<http://www.who.int/csr/ihr/en/index.html>, retrieved 13 December 2007). [↑](#footnote-ref-115)
